# Lipid-anchored proteasomes control membrane protein homeostasis

**DOI:** 10.1126/sciadv.adj4605

**Published:** 2023-11-29

**Authors:** Ruizhu Zhang, Shuxian Pan, Suya Zheng, Qingqing Liao, Zhaodi Jiang, Dixian Wang, Xuemei Li, Ao Hu, Xinran Li, Yezhang Zhu, Xiaoqi Shen, Jing Lei, Siming Zhong, Xiaomei Zhang, Lingyun Huang, Xiaorong Wang, Lan Huang, Li Shen, Bao-Liang Song, Jing-Wei Zhao, Zhiping Wang, Bing Yang, Xing Guo

**Affiliations:** ^1^ Zhejiang Provincial Key Laboratory for Cancer Molecular Cell Biology, Life Sciences Institute, Zhejiang University, Hangzhou 310058, China.; ^2^ National Institute of Biological Sciences, Beijing 102206, China.; ^3^ Tsinghua Institute of Multidisciplinary Biomedical Research, Tsinghua University, Beijing 100084, China.; ^4^ Department of Human Anatomy, Histology and Embryology, System Medicine Research Center, and Department of Pathology of Sir Run Run Shaw Hospital, Zhejiang University School of Medicine, Hangzhou 310058, China.; ^5^ Cryo-Electron Microscopy Center, Zhejiang University, Hangzhou 310058, China.; ^6^ Hubei Key Laboratory of Cell Homeostasis, College of Life Sciences, Taikang Center for Life and Medical Sciences, Taikang Medical School, Wuhan University, Wuhan 430072, China.; ^7^ Zhejiang University-Hangzhou Global Scientific and Technological Innovation Center, Hangzhou 311200, China.; ^8^ Department of Neurobiology and Department of Neurology of Second Affiliated Hospital, NHC and CAMS Key Laboratory of Medical Neurobiology, Zhejiang University School of Medicine, Hangzhou 310058, China.; ^9^ The MOE Frontier Science Center for Brain Research and Brain-Machine Integration, Zhejiang University School of Brain Science and Brain Medicine, Hangzhou 310058, China.; ^10^ Zhejiang University-University of Edinburgh Institute, Zhejiang University, Haining 314400, China.; ^11^ Deanery of Biomedical Sciences, College of Medicine and Veterinary Medicine, University of Edinburgh, Edinburgh EH8 9YL, UK.; ^12^ Department of Physiology and Biophysics, University of California-Irvine, Irvine, CA 92697, USA.; ^13^ Department of Developmental and Cell Biology, University of California-Irvine, Irvine, CA 92697, USA.

## Abstract

Protein degradation in eukaryotic cells is mainly carried out by the 26*S* proteasome, a macromolecular complex not only present in the cytosol and nucleus but also associated with various membranes. How proteasomes are anchored to the membrane and the biological meaning thereof have been largely unknown in higher organisms. Here, we show that *N*-myristoylation of the Rpt2 subunit is a general mechanism for proteasome-membrane interaction. Loss of this modification in the Rpt2-G2A mutant cells leads to profound changes in the membrane-associated proteome, perturbs the endomembrane system, and undermines critical cellular processes such as cell adhesion, endoplasmic reticulum–associated degradation and membrane protein trafficking. Rpt2^G2A/G2A^ homozygous mutation is embryonic lethal in mice and is sufficient to abolish tumor growth in a nude mice xenograft model. These findings have defined an evolutionarily conserved mechanism for maintaining membrane protein homeostasis and underscored the significance of compartmentalized protein degradation by myristoyl-anchored proteasomes in health and disease.

## Teaser

Membrane-based protein degradation dictates embryonic development and tumor growth.

## INTRODUCTION

Proteins in eukaryotic cells are highly compartmentalized, and so are their synthesis and degradation (*1*, *2*). Cells have evolved multiple strategies to dispatch proteins to different locations for degradation under normal or stressed conditions (*2*–*7*). This implies that the 26*S* proteasome, which digests the majority of eukaryotic proteins, must be available when substrates emerge or arrive. Consisting of a 20*S* core particle (CP) and one or two 19*S* regulatory particles (RPs), the 26*S* proteasome is highly abundant but not evenly distributed in cells. How the subcellular localization of these macromolecular complexes is determined is still not fully understood (*8*–*11*). In particular, a number of studies since the early 1990s have reported proteasomes that bound to various membrane structures of cells from different tissues and species (*11*). In some cases, this was attributed to proteasome binding to specific membrane proteins, whereas a general mechanism for proteasome-membrane association has been lacking, precluding further investigation of its biological meaning.

On the other hand, proteasomes are known to be regulated by a variety of chemical modifications including *N*-myristoylation (*12*), a cotranslational lipid modification on proteins that start with methionine and glycine (Met_1_-Gly_2_). After removal of the initiator Met, the myristoyl group can be covalently conjugated to Gly2 by *N*-myristoyltransferases 1 and 2 (NMT1/2) (*13*, *14*). In HeLa cells, scores of proteins were found to be modified this way, most of which are membrane-targeted (*15*). Secure membrane anchoring of myristoylated proteins requires additional modifications (e.g., palmitoylation) or electrostatic interaction between positively charged amino acids with the negatively charged head groups of phospholipids. In the latter case, membrane association can be antagonized by a phosphorylation event nearby (*16*, *17*). Since no endogenous demyristoylating enzymes have been identified, *N*-myristoylation is currently considered irreversible. Nonetheless, the bacterial effector protein IpaJ from *Shigella* can specifically cleave after the myristoylated glycine residue and cause a proteolytic loss of protein myristoylation in the host cell (*18*, *19*).

Rpt2/PSMC1, a component of the 19*S* RP, is the only proteasome subunit that begins with Met_1_-Gly_2_, thus the only subunit that can be *N*-myristoylated. Rpt2-Gly2 myristoylation has been detected in multiple mass spectrometry (MS) studies from yeast to plants to mammals (*15*, *20*–*24*), and the N-terminal MG motif of Rpt2 is invariant through evolution (fig. S1A). In yeast, Rpt2 myristoylation has been suggested to target proteasomes to the nuclear envelope (*25*), and deletion or mutation of the myristoylation site (Rpt2-ΔG or G2A) led to impaired quality control of nuclear proteins (*26*). In human cells, Rpt2 appears to be one of the most myristoylated proteins, similar to the well-established membrane-localized kinases such as Src and PRKACA (*15*). However, the function of Rpt2 myristoylation in higher eukaryotes has not been investigated, except for our recent finding that it is required for Rpt2-Tyr439 phosphorylation by Src (*27*).

Here, we have comprehensively characterized Rpt2 myristoylation-dependent proteasome association with the membrane in mammalian cells. Unlike in yeast, myristoyl-anchored proteasomes (MAPs) have broad subcellular distribution at various membrane structures and undergo different regulations. These membrane-associated proteasomes play a fundamental role in controlling endomembrane homeostasis, membrane protein trafficking, embryonic development, and tumorigenesis. These findings underscore the biological significance of compartmentalized protein degradation in higher organisms and suggest a potential direction for translational research through selective targeting of the membrane-tethered proteasomes.

## RESULTS

### Rpt2 myristoylation anchors proteasomes to the membrane in mammalian cells

**Fig. 1.**
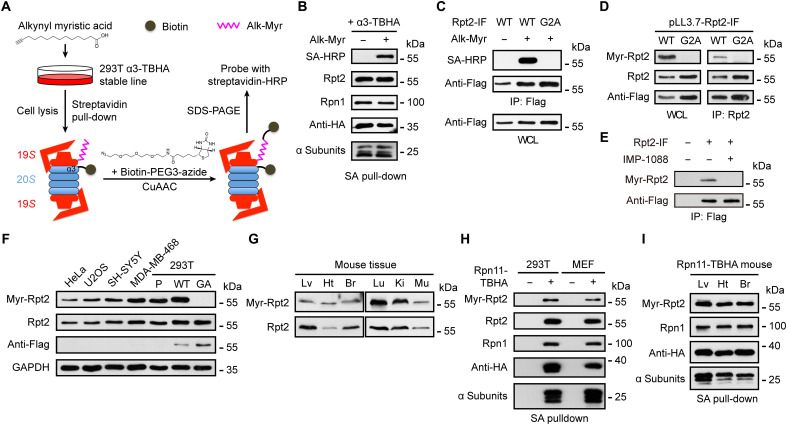
Rpt2 myristoylation is widely present across different mammalian cells and tissue types. (**A**) Procedure of detecting endogenous Rpt2 myristoylation by metabolic labeling and click chemistry. CuAAC, copper(I)-catalyzed azide-alkyne cycloaddition. (**B**) Proteasome samples purified by streptavidin (SA) pull-down were subjected to click chemistry reaction as in (A) and probed with SA–horseradish peroxidase (HRP) and the indicated antibodies. (**C**) 293 T cells were transfected with the indicated Rpt2-IF constructs (“internal Flag”) (*27*) and treated with or without Alk-Myr. After anti-Flag immunoprecipitation, Rpt2 myristoylation was detected by click chemistry. (**D**) 293 T cells were stably transduced with pLL3.7-Rpt2-IF [wild type (WT) or G2A] for knockdown of endogenous Rpt2 and simultaneous expression of the Rpt2-IF variants. Whole-cell lysates (WCLs) and anti-Rpt2 immunoprecipitates were probed with the indicated antibodies. (**E**) 293 T cells were transfected with or without Rpt2-IF (WT). Dimethyl sulfoxide or IMP-1088 (1 μM) was added upon transfection. Cells were lysed after 12 hours and newly synthesized (transfected) Rpt2-IF was immunoprecipitated and probed with the indicated antibodies. (**F**) Western blot detection of Rpt2 myristoylation from the indicated cell lines. P, untransduced (parental) 293 T cells. “WT” and “GA” were 293 T cells stably transduced with pLL3.7-Rpt2-IF (WT or G2A) as in (D), used as control. (**G**) Straight Western blot detection of Rpt2 myristoylation from the indicated mouse tissues. Lv, liver; Ht, heart; Br, brain; Lu, lung; Ki, kidney; Mu, muscle. (**H**) Western blot detection of Rpt2 myristoylation from proteasomes affinity-purified from 293 T cells stably expressing Rpn11-TBHA or from Rpn11TBHA/TBHA mouse embryonic fibroblasts (MEFs). 293 T parental cells and WT MEFs were respectively used as control (“−”). (**I**) Immunoblot of myristoylated Rpt2 from proteasomes purified from the liver (Lv), heart (Ht), and brain (Br) tissues of Rpn11-TBHA mice.

We confirmed Rpt2 myristoylation in mammalian cells and its presence in the assembled proteasomes using two methods of detection. First, a clickable analog of myristic acid, Alk-Myr (*15*), was used to metabolically label 293 T cells stably expressing TBHA-tagged α3, a 20*S* subunit (Fig. 1A). The TBHA tag contains a biotinylation sequence and a hemagglutinin (HA) tag that facilitate the isolation and detection of proteasomes (*28*). Streptavidin-purified proteasome complexes from these cells were subjected to an in vitro click reaction, and myristoylated endogenous Rpt2 could be readily detected from the 26*S* proteasomes only in the presence of Alk-Myr (Fig. 1, B and C). Second, we developed a myristoylation-specific antibody against the N terminus of Rpt2, which detected myristoylation of Rpt2 in mammalian cells but not unmodified Rpt2 recombinantly expressed from *Escherichia coli* (fig. S1B). The immunoreactivity was completely eliminated by the G2A mutation (Fig. 1D) or by overexpression of IpaJ (fig. S1C). Rpt2 myristoylation was also abrogated by IMP-1088, a specific inhibitor NMT1/2 (*29*), and greatly diminished by tris(dibenzylideneacetone) dipalladium, a generic NMT1 inhibitor (Fig. 1E and fig. S1D) (*30*), further confirming the specificity of our detection methods. *N*-myristoylated Rpt2 was widely seen in total extracts from all cell/tissue types examined and on proteasome complexes affinity-purified from different organs of the Rpn11-TBHA knock-in mice (Fig. 1, F to I) (*31*). Sucrose gradient ultracentrifugation and native gel electrophoresis assays both showed that *N*-myristoylated Rpt2 was primarily present in fully assembled 26*S*/30*S* proteasomes (figs. S1E and S3D), consistent with its coprecipitation with the 20*S* CP (Fig. 1B). Together, these characterizations demonstrate that Rpt2 myristoylation occurs in mammalian cells and on the proteasome holoenzyme.

**Fig. 2.**
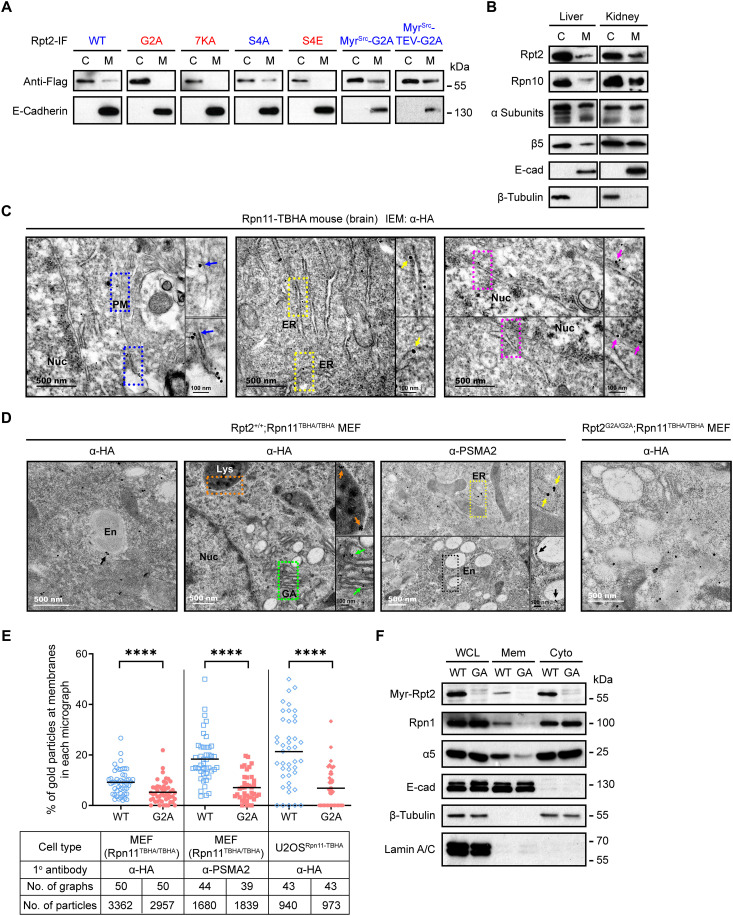
Rpt2 myristoylation mediates proteasome-membrane association. (**A**) 293 T cells were transfected with the indicated Rpt2-IF variants. After cell fractionation, cytosolic (C) and membrane (M) samples were probed. Rpt2-IF variants capable of membrane-binding were labeled blue, whereas nonmembrane-binding mutants were labeled red. 7KA, K15/16/19/21/22/23/24A. E-Cadherin marks the membrane fractions. (**B**) Immunoblot of proteasome subunits in cytosolic (C) and membrane (M) fractions of mouse tissues. (**C**) Representative immunoelectron microscopy (IEM) images of cerebral cortex slices from Rpn11^TBHA^ mice stained with an anti-HA antibody (scale bars, 500 nm). Boxed areas are enlarged and shown on the right, with membrane-localized gold particles indicated by arrows of colors corresponding to different membranes (scale bars, 100 nm). PM, plasma membrane; ER, endoplasmic reticulum; Nuc, nucleus. (**D**) Representative IEM images of the indicated MEF cells stained with anti-HA or anti-PSMA2 antibodies. En, endosome; Lys, lysosome; GA, Golgi apparatus. (**E**) Quantified results of IEM experiments performed with the indicated cell types and antibodies. Each data point represents an independent micrograph. Lines indicate the means of each group. ****P < 0.0001 (Mann-Whitney test, two-tailed). (**F**) Membrane (Mem) and cytosolic (Cyto) fractions as well as WCLs of WT and G2A (GA) MEFs were probed with the indicated antibodies. Lamin A/C was used as a nuclear protein marker.

As we have seen before (*27*), *N*-myristoylation is essential for Rpt2 association with the membrane since the Rpt2-G2A mutant was completely absent from membrane fractions of the cell (Fig. 2A). Fusing the myristoylation sequence of Src (amino acids 1 to 14) to the N terminus of Rpt2-G2A (Myr^Src^-Rpt2-G2A) restored membrane association of the latter without affecting its assembly into the proteasome (Fig. 2A and fig. S2A), confirming the specific role of the myristoyl group in Rpt2 membrane anchoring. It should be noted, though, that like many myristoylated proteins with an N-terminal electrostatic switch, membrane binding of wild-type (WT) Rpt2 is also dependent on a stretch of basic residues (K15/16/19/21/22/23/24) conserved through evolution (fig. S1A). Mutation of these lysines to alanine (7KA) inhibited Rpt2-membrane association (Fig. 2A). Conversely, the electrostatic interaction between those lysine residues and phospholipids could be disrupted by a phosphomimetic S4E mutation, but not the phospho-deficient S4A mutation (Fig. 2A). This result suggests that phosphorylation of Ser^4^ near the N terminus, which is one of the most frequently detected proteasome phosphosites conserved in vertebrates (fig. S1A) (*31*), may negatively regulate membrane localization of Rpt2.

To determine whether membrane binding of the proteasome complex is dependent on Rpt2 myristoylation in mammalian cells, we first confirmed by mouse tissue fractionation that, in addition to Rpt2, other RP and CP subunits of the proteasome were also present in the membrane fractions (Fig. 2B). We then took advantage of our Rpn11-TBHA knock-in mice and optimized an immunogold electron microscopy protocol to detect endogenous 26*S* proteasomes with anti-HA antibodies in cerebral cortex sections. The results further demonstrated the presence of proteasomes in not only the plasma membrane but also the nuclear envelope, endoplasmic reticulum (ER), and mitochondria of neurons (Fig. 2C and fig. S2B).

Next, we obtained Rpt2^+/+^;Rpn11^TBHA/TBHA^ and Rpt2^G2A/G2A^;Rpn11^TBHA/TBHA^ mouse embryonic fibroblasts (MEFs) by crossing Rpn11^TBHA/+^ mice with Rpt2^G2A/+^ mice (fig. S3E). Immunoelectron microscopy (IEM) with anti-HA and anti-PSMA2 (a 20*S* subunit) antibodies on WT MEFs (Rpt2^+/+^;Rpn11^TBHA/TBHA^) showed proteasome localization at additional membranes including the endosome, lysosome, and the Golgi apparatus (Fig. 2D), consistent with several earlier reports (*32*–*34*). Overall, approximately 5 to 10% of gold particles were found to be membrane-localized from all the micrographs that contained evident membranous structures. Membrane association of the proteasome was significantly reduced in the Rpt2^G2A/G2A^ MEFs (Fig. 2E). We also applied anti-HA IEM on U2OS cells stably expressing Rpn11-TBHA (fig. S2B). Similar to the MEF results, membrane-localized proteasomes were significantly decreased in U2OS^G2A/G2A^ cells generated by CRISPR-Cas9 (Fig. 2E). Further analysis of the IEM results revealed that loss of Rpt2 myristoylation mainly interfered with proteasome localization at the plasma membrane, ER, Golgi apparatus, and endosomes/lysosomes (“PERGEL”), whereas mitochondria- and nuclear envelope–associated proteasomes (“MN”) were less affected (fig. S2C). This pattern agrees well with the global distribution of myristoylated proteins identified by proteomics (*15*) and suggests that Rpt2 myristoylation can direct the proteasome to various subcellular locations. Moreover, cell fractionation assays also demonstrated a clear decrease of 19*S* and 20*S* components in the membrane fraction from Rpt2-G2A cells compared to the control (Fig. 2F). Last, using total internal reflection fluorescence imaging with structured illumination microscopy (TIRF-SIM), we observed distinct signals of green fluorescent protein (GFP)–tagged Rpn10 (another 19*S* subunit) in the close vicinity of the ventral plasma membrane in WT cells, but much less so in G2A knock-in cells (fig. S2D). Together, these results indicate that Rpt2 *N*-myristoylation is a major and prevalent mechanism mediating proteasome-membrane association. We hereby refer to these myristoyl-anchored proteasomes as MAPs.

**Fig. 3.**
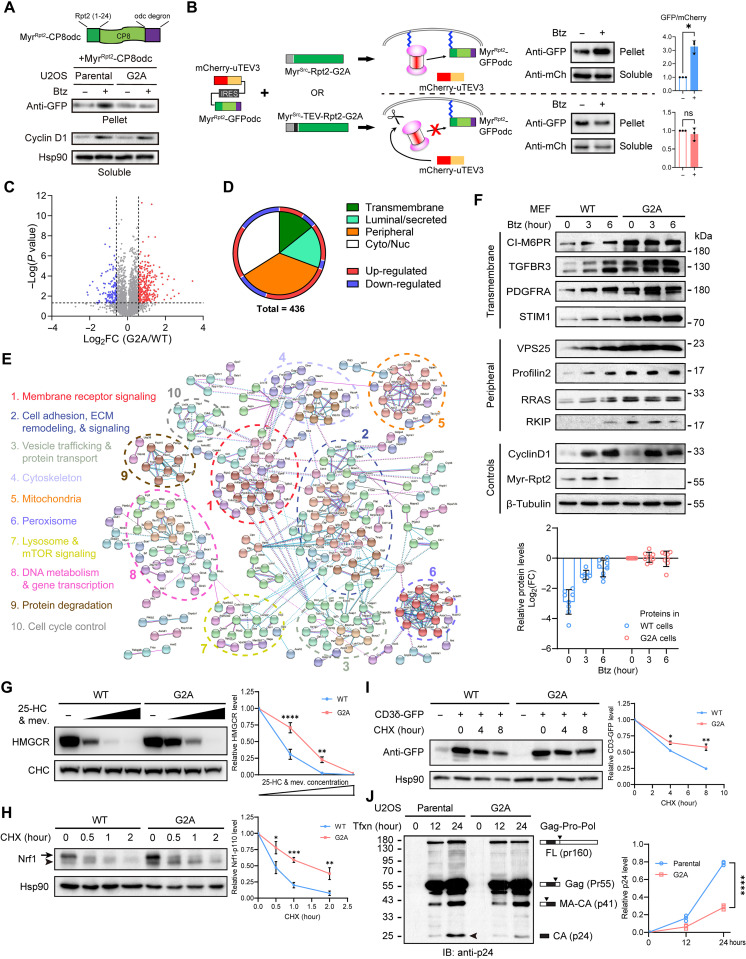
Loss of myristoylation interferes with membrane protein turnover and processing. (**A**) Single clones of U2OS cells stably expressing the Myr^Rpt2^-CP8odc reporter (top) were treated with bortezomib (Btz; 5 μM, 3 hours). Membrane (pellet) and soluble fractions were analyzed by immunoblotting. (**B**) Left: The cleavable Rpt2 system for assessing membrane-associated proteasome activity. After Btz treatment, Myr^Rpt2^-GFPodc was immunoblotted and quantified (right). *P < 0.05 (Student’s *t*-test, two-tailed, *N* = 3). ns, not significant. (**C**) Volcano plot of SILAC-MS results from MEFs. (**D**) Summary of differentially regulated proteins in G2A cells and their subcellular locations. (**E**) Protein-protein interaction network of up-regulated proteins in G2A MEFs. (**F**) WT and G2A MEFs were treated with Btz (1 μM) and probed for representative proteins. The abundance of each protein (circles) was quantified after normalization to their basal levels in G2A cells (bottom). (**G**) WT or G2A MEFs were treated with increasing concentrations of 25-hydroxycholesterol (25-HC) and mevalonate (mev) for 4 hours and probed for HMGCR (3-hydroxy-3-methylglutaryl–coenzyme A reductase). CHC (clathrin heavy chain) was shown as a loading control. **P < 0.01 and ****P < 0.0001 (Student’s *t* test, two-tailed, *N* = 3). (**H**) Cells were treated with cycloheximide (CHX; 50 μg/ml) and probed for Nrf1. Arrow, full-length (FL) Nrf1. Arrowhead, the p110 fragment. *P < 0.05, **P < 0.01, and ***P < 0.001 (Student’s *t* test, two-tailed, *N* = 4). (**I**) CD3δ-GFP was transiently expressed in U2OS cells and immunoblotted after treatment with CHX. *P < 0.05, and **P < 0.01 (Student’s *t* test, two-tailed, *N* = 3). (**J**) Cells were transfected with FL Gag-Pro-Pol from the psPAX2 plasmid and harvested at the indicated time after transfection (tfxn). Downward triangles indicate major cleavage sites. MA, matrix protein; CA, capsid protein. Arrowhead, the final product (CA/p24). ****P < 0.0001 (Student’s *t* test, two-tailed, *N* = 3).

To specifically detect the activity of MAPs toward membrane-bound protein substrates, we fused the Rpt2 N-terminal sequence (amino acids 1 to 24 including the myristoylation site and the polybasic region) (*27*) to a commonly used model substrate of the proteasome, GFPodc (*35*), or its loosely folded variant CP8odc (Fig. 3A and fig. S2E) (*36*). The resulting membrane-targeted Myr^Rpt2^-CP8odc reporter was stabilized by the proteasome inhibitor bortezomib (Btz) in the membrane fraction of WT cells, suggesting that it underwent constant local proteasomal degradation (Fig. 3A). However, membrane-bound Myr^Rpt2^-CP8odc showed no response to Btz in the G2A cells (Fig. 3A), consistent with the lack of proteasome activity at the membrane and also implying that cytosolic proteasomes in G2A cells cannot efficiently degrade this membrane-localized substrate. Btz-induced accumulation of nonmembrane substrates (e.g., cyclin D1) was not affected by the G2A mutation (Fig. 3A). As these results were obtained from single clones of cells stably expressing the reporter protein, we wanted further confirmation by acutely disrupting proteasome-membrane interaction in a cell population. To this end, a cleavable version of Myr^Src^-Rpt2-G2A was engineered by inserting the tobacco etch virus (TEV) protease recognition motif (ENLYFQ|S) behind the Myr^Src^ sequence. The resulting Myr^Src^-TEV-Rpt2-G2A could associate with the membrane just like Myr^Src^-Rpt2-G2A but could be cut and released from the membrane upon TEV protease expression (Fig. 2A and fig. S2, A and F). We knocked down endogenous Rpt2 in WT cells, simultaneously expressed either of these two forms of Rpt2, and then cointroduced Myr^Rpt2^-GFPodc together with uTEV3, an optimized version of the TEV protease (Fig. 3B) (*37*). Btz treatment of cells expressing Myr^Src^-Rpt2-G2A (noncleavable) led to the accumulation of Myr^Rpt2^-GFPodc as expected. In contrast, this reporter was insensitive to Btz treatment in cells expressing Myr^Src^-TEV-Rpt2-G2A, in which the Rpt2 protein had been dissociated from the membrane by uTEV3 (Fig. 3B). This latter result is very similar to that in G2A knock-in cells (Fig. 3A). Such acute perturbation of membrane binding further demonstrates the direct contribution of MAPs to compartmentalized protein degradation at the membrane.

Despite the local effect, loss of Rpt2 myristoylation did not affect overall proteasomal abundance, activity, or cell viability (fig. S3, A to C). Proteasome holoenzyme assembly remained intact in Rpt2-G2A cells as judged by native gel electrophoresis (fig. S3D) and pull-down MS experiments (fig. S3, E and F). However, dislodgement of the G2A proteasome from the membrane considerably altered its interactome. Nearly 500 proteasome-interacting proteins (PIPs) (*23*) were identified in both WT and G2A cells from two independent MS experiments, over a third of which showed enhanced association with the G2A proteasome. These proteins are located throughout the cell, mostly in the nucleus and cytoplasm (fig. S3F and table S1). On the other hand, a few PIPs exhibited reduced proteasome binding in the G2A cells. These included the membrane-localized tyrosine phosphatase PTPN2 and tyrosine kinase Src, which together regulate Rpt2-Y439 phosphorylation and membrane proteasome function as we recently reported (fig. S3, E and F) (*27*). Also reduced/lost in the G2A proteasome interactome was the nonessential proteasome chaperone, Ecm29 (fig. S3F), which is known to be located at membrane-bound organelles such as endosomes and the ER (*32*, *38*). Therefore, blocking Rpt2 myristoylation may repartition proteasomes between the membrane and soluble compartments, potentially leading to proteomic and functional alterations of the cell.

### MAPs are required for the proper turnover and processing of membrane-related proteins

To gain insights into the proteins selectively controlled by MAPs, we performed quantitative MS on total cell extracts of WT and G2A MEFs with reciprocal SILAC labeling (stable isotope labeling using amino acids in cell culture). A total of 6774 proteins were detected from three biological repeats, with 4470 proteins identified in all three runs (Fig. 3C, fig. S4A, and table S2). Nearly one-tenth of these proteins (436) were differentially regulated in G2A cells [|Log_2_FoldChange(G2A/WT)| > 0.585], with 66.6% of them being membrane-/organelle-associated based on annotations from UniProt and the Human Protein Atlas (Fig. 3D). These include peripheral membrane proteins, transmembrane proteins, and luminal/secreted proteins, which are widely localized at various subcellular sites and involved in a variety of biological functions (Fig. 3, D and E, fig. S4, B and C, and tables S3 and S4). Overall, there was a minimal correlation between their protein and transcript levels (*r*^2^ = 0.099), agreeing with a posttranslational mechanism underlying their differential expression (fig. S4D). Of note, the majority of these proteins showed increased abundance within G2A cells (Fig. 3, C to E, and fig. S4B), and the up-regulation of multiple transmembrane and peripheral proteins was further confirmed by immunoblotting (Fig. 3F and fig. S4E). In addition to membrane association, these up-regulated proteins also shared the following features: (i) They became accumulated in WT cells following Btz treatment; (ii) in G2A cells, however, they remained high and exhibited no or only minor responses to Btz (Fig. 3F and fig. S4F); (iii) as noted above, the mRNA expression of most of these proteins was comparable between WT and G2A cells (fig. S4E); (iv) their elevated protein levels could be reversed by expressing the membrane-targeted Myr^Src^-Rpt2-G2A in the G2A cells (fig. S4G). Moreover, at least some of these proteins were more evidently polyubiquitinated via the K48 linkage in G2A cells than in WT cells, even without proteasome inhibitor treatment (fig. S4H). These data indicate that MAPs are uniquely responsible for the turnover of a subset of proteins with diverse subcellular localizations and functions, and such turnover is largely blocked in cells lacking Rpt2 myristoylation.

An important mechanism for membrane protein regulation and quality control is through ER-associated degradation (ERAD), dysregulation of which usually leads to ER stress and unfolded protein responses (*39*, *40*). ERAD substrates are generally believed to be delivered to cytosolic proteasomes for degradation, while we wondered if MAPs also participate in this process. Our MS results showed that not all ERAD substrates (*40*, *41*) accumulated in G2A MEFs, and no clear signs of ER stress were observed in the mutant cells, either (fig. S5, A to G). This was expected given the topological and biochemical complexity of the ERAD system (*42*, *43*). We then specifically assessed the turnover of three well-recognized ERAD substrates in WT and G2A cells. First, HMGCR (3-hydroxy-3-methylglutaryl–coenzyme A reductase), the rate-limiting enzyme in cholesterol synthesis, can be rapidly degraded via ERAD (*44*). Sterol stimulation triggered HMGCR degradation in a dose-dependent manner in WT MEFs. In G2A MEFs, however, this effect was clearly weakened (Fig. 3G). Similarly, degradation of the ER-resident transcription factor Nrf1/NFE2L1 was also less efficient in G2A cells, resulting in a prolonged presence of the truncated p110 fragment of Nrf1/NFE2L1 (*45*, *46*) (Fig. 3H). The third example was CD3δ. When not assembled into the T cell receptor complex, ectopically expressed CD3δ is known to be cleared by ERAD (*47*), which was again compromised in G2A cells (Fig. 3I). These findings demonstrate that at least some ERAD substrates rely on the MAPs for efficient degradation.

We also examined a pathologically relevant protein lipid-anchored to the plasma membrane, the Gag protein of HIV. It is well established that *N*-myristoylation and plasma membrane association of Gag is critical for its processing, viral particle assembly, and virus budding (*48*, *49*). Proteolysis and maturation of Gag from HIV and multiple other retroviruses also depend on proteasome activity (*50*, *51*). We expressed the full-length Gag-Pro-Pol protein in U2OS cells and monitored the processing of Gag over time. The 55-kDa Gag protein emerged similarly in control and G2A cells, but subsequent production of the p24 fragment (capsid protein, CA), which depends on membrane binding, was much delayed in U2OS-G2A cells (Fig. 3J). This effect echoes previous results obtained with proteasome inhibitors (*50*) and suggests a specific requirement for MAPs in viral protein processing.

### Endomembrane homeostasis and protein trafficking are dysregulated in cells lacking MAPs

**Fig. 4.**
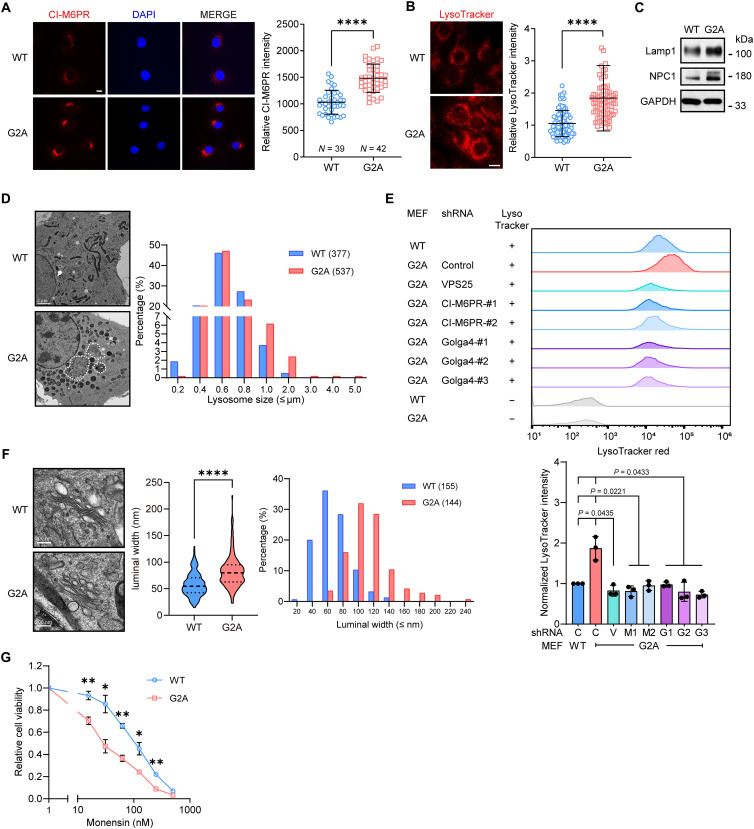
Perturbation of the endomembrane system in Rpt2-G2A cells. (**A**) Immunofluorescence staining of CI-M6PR in G2A MEFs. Scale bar, 10 μm. ****P < 0.0001 (two-tailed *t* test, unpaired). (**B**) LysoTracker staining of MEFs. Scale bar, 10 μm. ****P < 0.0001 (two-tailed *t* test, unpaired. *N* = 88 each). (**C**) Western blot analysis of lysosome-related proteins in MEFs. (**D**) Representative transmission electron microscopy (TEM) images showing lysosomes/multivesicular bodies in WT and G2A MEFs (left). Lysosome sizes were measured and quantified (right). Abnormally large, membrane-bound vacuoles (dotted lines) were occasionally observed in G2A cells but not in WT cells. Scale bars, 2 μm. (**E**) MEFs were transduced with the indicated short hairpin RNAs (shRNAs) and stained with LysoTracker. Representative histograms of flow cytometry analysis are shown on top. Quantified results from three independent experiments are at the bottom [repeated-measures one-way analysis of variance (ANOVA), with Geisser-Greenhouse corrections]. C, control shRNA; V, VPS25; M, CI-M6PR; G, Golga4. (**F**) Representative TEM images of the Golgi apparatus in WT and G2A MEFs. The luminal width of individual Golgi cisterna was measured and quantified. ****P < 0.0001 (Welch’s *t* test). Scale bars, 200 nm. (**G**) WT and G2A MEFs were treated with increasing concentrations of monensin for 48 hours and released for another 24 hours. Cell viability was measured. *P < 0.05 and **P < 0.01 (two-tailed *t* test, paired, *N* = 3).

Also differentially expressed in G2A cells were numerous proteins implicated in organellar function and intracellular trafficking (Fig. 3E). For instance, cation-independent mannose 6-phosphate receptor (CI-M6PR; also known as Igf2r), a key mediator of protein sorting between Golgi and lysosomes/late endosomes, and its Golgi-resident receptor Golga4/Golgin-245 (*52*, *53*) were both up-regulated in G2A MEFs and could be returned to normal levels upon Myr^Src^-Rpt2-G2A expression (Figs. 3, E and F, and 4A and fig. S4, E and G). In addition, vacuolar protein sorting–associated protein 25 (VPS25) and its binding partner VPS36 were elevated in G2A cells (Fig. 3, E and F), both of which are central components of the ESCRT-II complex (endosomal sorting complex required for transport II) required for sorting of endosomal cargo proteins into multivesicular bodies then lysosomes (*54*). These findings prompted us to examine the relevant compartments. Compared to WT control, G2A cells exhibited significantly stronger LysoTracker staining with a concurrent stabilization of lysosomal proteins (Fig. 4, B, C, and E, and fig. S6, A and B). Transmission electron microscopy (TEM) also showed an increase in the number of lysosomes/MVBs in G2A cells. Although their overall sizes were comparable to those in WT cells, we occasionally observed extraordinarily large, membrane-bound vacuoles/inclusions in G2A cells that were not seen in WT cells (Fig. 4D). Partial depletion of CI-M6PR, Golga4, or VPS25 using short hairpin RNAs (shRNAs) effectively reduced LysoTracker signals in the mutant cells (Fig. 4E and fig. S6, C to E). Golgi cisternae, on the other hand, appeared swollen in G2A cells as revealed by TEM. The mean luminal width of individual cisterna in G2A cells (82.8 nm) was significantly larger than that in control cells (58.0 nm) (Fig. 4F). The G2A MEFs were also more sensitive to the Golgi stress inducer, monensin (*55*) (Fig. 4G), indicating impairment of Golgi function.

**Fig. 5.**
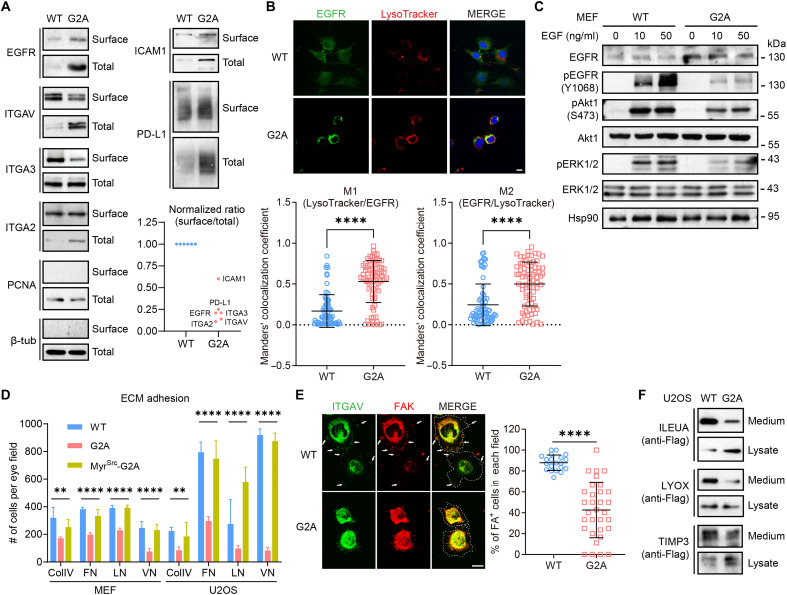
Cell surface protein expression is compromised in the absence of MAPs. (**A**) Cell surface biotinylation assay of the indicated proteins. Each blot is representative of at least three independent experiments. The surface/total ratio of each protein was normalized to that in WT cells. EGFR, integrins, and proliferating cell nuclear antigen (PCNA) results were from MEFs. ICAM-1, PD-L1, and β-tubulin results were from U2OS cells. PCNA and β-tubulin were shown as controls for the specificity of surface labeling. (**B**) Costaining of MEFs with anti-EGFR antibody and LysoTracker. Scale bar, 10 μm. Mander’s colocalization coefficients between the two channels were calculated for both cell types; ****P < 0.0001 (unpaired two-tailed *t* test. WT, *N* = 72; G2A, *N* = 76.) (**C**) MEFs were serum-starved for 24 hours and treated with different concentrations of EGF for 5 min. Total cell lysates were blotted. (**D**) Quantified results of cell adhesion assays with WT and G2A MEFs on surfaces coated with different ECM proteins. ColIV, collagen IV; FN, fibronectin; LN, laminin; VN, vitronectin. **P < 0.01 and ****P < 0.0001 (*N* = 18 eye fields from three independent experiments; one-way ANOVA). (**E**) WT and G2A MEFs were seeded on fibronectin-coated coverslips for 30 min and immunostained with the indicated antibodies. Dotted lines mark cell contours. Arrows indicate peripheral focal adhesion (FA)–like structures [double positive for ITGAV and FA kinase (FAK)]. Scale bar, 10 μm. ****P < 0.0001 (two-tailed *t* test, unpaired). WT, *N* = 20 eye fields (318 cells); G2A, *N* = 32 eye fields (179 cells). (**F**) C-Terminally Flag-tagged expression constructs of the indicated secreted proteins were transfected into U2OS cells. Cell media were immunoprecipitated with anti-Flag antibodies and probed together with each corresponding WCL.

To understand whether such perturbation of the endomembrane system could lead to defects in protein trafficking, we examined the cell surface expression of several classic transmembrane proteins including epidermal growth factor receptor (EGFR), intercellular adhesion molecule–1 (ICAM-1), programmed death-ligand 1 (PD-L1), and integrins (ITGA2/3/V) by surface biotinylation assays. Most of these proteins showed a higher total level in G2A cells. In contrast, all of them had a much lower surface/total ratio, meaning that a larger proportion of these proteins failed to reach or maintain at the plasma membrane in the absence of MAPs (Fig. 5A). Immunofluorescence staining demonstrated that the majority of EGFR in G2A MEFs was inside the cells, with a significantly higher fraction colocalizing with lysosomes than observed in WT cells (Fig. 5B). Functionally, the G2A cells responded poorly to EGF despite the higher total level of EGFR, and downstream activation of Akt and Erk1/2 were markedly undermined (Fig. 5C).

Reduced surface presentation of integrin molecules predicts weakened cell adhesion, which is consistent with our proteomic and transcriptomic analyses (Fig. 3E and figs. S4C and S7A) and our routine observation that G2A knock-in cells were easier to dissociate from the vessels by trypsinization than their WT counterparts (fig. S7, B and C). Using defined extracellular matrix (ECM) components, we found that attachment of G2A cells to collagen IV-, fibronectin-, laminin- or vitronectin-coated surfaces was all significantly weaker than control, in agreement with attenuation of integrin function (Fig. 5D and fig. S7D). Upon seeding onto ECM-coated surface, WT cells readily spread out and formed focal adhesion–like structures positively stained with FAK and ITGAV at cell periphery, while this occurred much less efficiently in G2A cells (Fig. 5E). The weakened adhesion of G2A cells could be rescued by expression of Myr^Src^-Rpt2-G2A (Fig. 5D and fig. S7D), strongly suggesting that the adhesion defect of G2A cells resulted from the lack of MAPs. As expected, cell migration was also severely undermined by the Rpt2-G2A mutation in both wound-healing/scratch assays and transwell migration assays (fig. S7, E and F).

Furthermore, secretion of certain extracellular proteins was also impeded by the G2A mutation (Fig. 5F). Such intracellular entrapment may explain their apparent “up-regulation” in G2A MEFs as identified by MS (Fig. 3E). Together, these data demonstrate that MAPs play a fundamental role in maintaining the homeostasis of the endomembrane system, thereby having a broad impact on protein trafficking along the secretory pathway.

### MAPs are essential for tumorigenesis and embryonic development

**Fig. 6.**
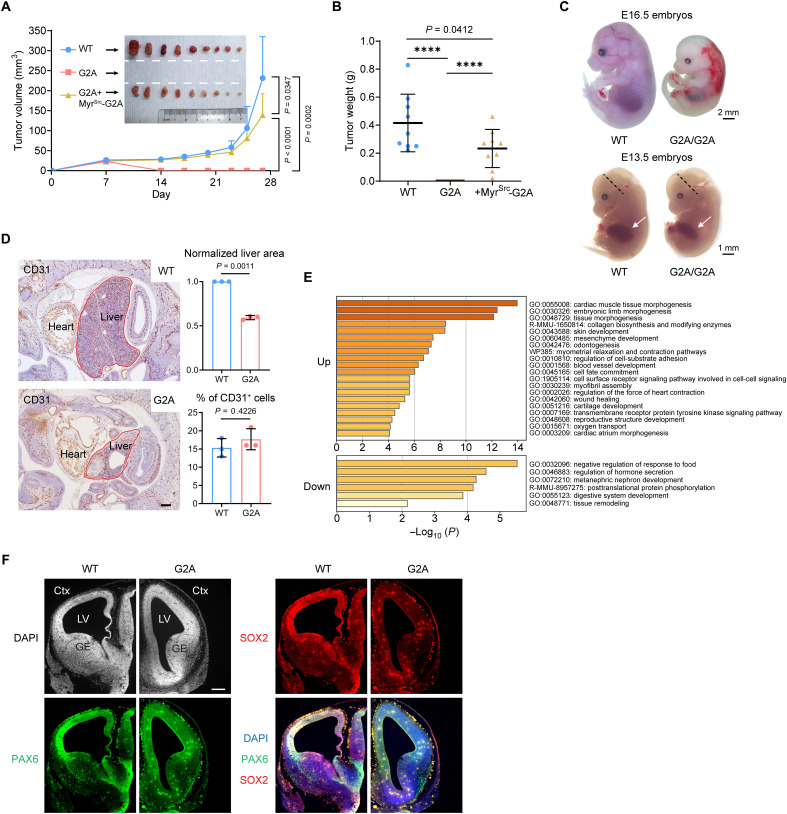
Proteasome myristoylation is required for tumorigenesis and embryonic development. (**A** and **B**) The indicated transformed MEFs were subcutaneously grafted into nude mice, and the sizes of xenograft tumors were monitored (A). Dissected tumors were photographed [inset of (A)] and weighed (B). ****P < 0.0001, Welch’s *t* test, *N* = 9. (**C**) Representative images of WT and G2A/G2A mouse embryos (littermates) at E16.5 (top) and E13.5 (bottom). Arrows point to the fetal liver. The dotted lines indicate the positions of the coronal sections shown in (F). (**D**) Immunohistochemistry staining of E13.5 embryos. Scale bar, 200 μm. Outlines of the liver area are marked, and anti-CD31 stains blood vessels. Normalized liver sizes and the percentage of CD31^+^ cells in each photograph were quantified (two-tail paired *t* test, *N* = 3 pairs of littermates). (**E**) Gene Ontology (GO) term analysis of differentially expressed mRNAs in G2A fetal livers (E12.5) as determined by RNA sequencing. (**F**) Immunohistochemistry staining of E13.5 mouse brain. Corresponding coronal sections of WT and G2A embryos [as shown in (C)] are juxtaposed for direct comparison. LV, lateral ventricle; GE, ganglionic eminence; Ctx, cortex; DAPI, 4′,6-diamidino-2-phenylindole. Scale bar, 200 μm.

Membrane receptor signaling, cell adhesion, protein secretion, and vesicular trafficking are all instrumental for oncogenesis. The ESCRT-II complex and CI-M6PR have also been suggested to be tumor suppressors (*52*, *56*). To demonstrate the consequences of the above cellular changes in vivo, we adopted a xenograft tumor model. The immortalized MEFs (WT and G2A) were further transformed with hTERT/N-Ras^G12V^ (*57*) and implanted subcutaneously into nude mice (fig. S8A). Oncogene-transformed WT cells readily formed tumors as expected. In sharp contrast, the MEFs of G2A origin completely failed in tumorigenic growth (Fig. 6, A and B). In tissue culture, however, the transformed G2A MEFs showed no growth defects (they grew slightly better than WT MEFs; fig. S8B). This indicates that the inability of the G2A cells to grow as xenografts was probably due to anomalous interactions with their microenvironment in vivo, as can be deduced from the in vitro studies above. Again, the replacement of endogenous Rpt2-G2A with Myr^Src^-Rpt2-G2A in transformed G2A MEFs largely restored the tumorigenicity of the cells (Fig. 6, A and B), highlighting the critical role of MAPs in tumorigenesis.

Moreover, homozygous Rpt2-G2A mutation in mice caused embryonic lethality with 100% penetrance (Fig. 6C). The G2A/G2A embryos died before E16.5, showing hemorrhage at the dorsal and interdigital regions with other parts of the body being anemic (Fig. 6C). In mutant embryos at earlier stages (e.g., E12.5 to 13.5), severe developmental defects of vital organs such as liver and brain could be detected by histology (Fig. 6, C to G). Not only the fetal liver was much smaller in the G2A embryos but also the lobule-like organization of liver cells was also largely absent (Fig. 6D). Transcriptomic analysis of mutant fetal livers identified a small fraction of genes with aberrant expression, which are enriched in essential development-related activities including cell adhesion, tissue morphogenesis, and cell fate determination (Fig. 6E). The mutant brain also exhibited abnormal morphology. PAX6^+^ and SOX2^+^ neural progenitor/stem cells were greatly reduced in number and showed distinct distributions from those in WT embryos, indicative of delayed neurogenesis and impaired cell migration (Fig. 6F). The highest level of Rpt2 myristoylation in the brain was seen at the pre- and perinatal stages, mirroring its indispensable role during brain development (fig. S8C). Together, these phenotypes provide strong in vivo evidence for the physiological significance of MAPs in mammals, which is well in line with the strict conservation of Rpt2 myristoylation through evolution.

## DISCUSSION

Among several mechanisms through which the proteasome can be targeted to the membrane (*11*), our results favor Rpt2 myristoylation as a major determinant of proteasome-membrane interaction. This has allowed us to interrogate the biological meaning of membrane-localized proteasomes by studying the Rpt2-G2A mutant. Distinct from earlier studies in yeast, where Rpt2 myristoylation directs proteasomes to the nuclear membrane (*25*, *26*), we have shown that the same modification in mammalian cells is responsible for broadly targeting 26*S* proteasomes to the plasma membrane and various organelles. In addition, membrane association of mammalian proteasomes can be antagonized by phosphorylation of Rpt2-Ser^4^, a site that does not exist in yeast Rpt2 (fig. S1A). These observations suggest that higher organisms have evolved additional mechanisms for controlling localized protein degradation by MAPs, which is essential for cell-cell communications.

Consistent with the earlier proteomic result that Rpt2 is one of the most heavily *N*-myristoylated proteins in human cells (*15*), our ongoing research suggests that the overall stoichiometry of Rpt2 myristoylation seems quite high across different cell types. This may be attributed to the cotranslational and irreversible nature of such modification. Most, if not all, of myristoylated Rpt2 is incorporated into the 26*S* proteasome holoenzymes (figs. S1E and S3D). Contrary to these observations, however, only a small percentage of proteasomes were localized to the membrane (Fig. 2), and the majority of myristoylated Rpt2 was actually present in the cytosolic (soluble) fraction (Fig. 2F). Such discrepancy indicates that Rpt2 myristoylation alone is not sufficient for anchoring proteasomes to the membrane and that the hydrophobic myristoyl group on nonmembrane-localized Rpt2 must be somehow shielded from the aqueous environment. This would constitute a “myristoyl switch” that has been described for several myristoylated proteins (*18*, *19*, *58*–*63*), such that the myristoyl group is buried until a conformational change triggers its exposure and subsequent membrane association of the attached protein. Therefore, the amount of membrane-bound MAPs in a cell may be collectively determined by a number of factors including (i) the level of Rpt2 myristoylation, which does change (e.g., during brain development) via unknown mechanisms; (ii) Rpt2-Ser^4^ phosphorylation; (iii) possibly other membrane-localized PIPs; and (iv) unidentified factors that can mask/unmask Rpt2 myristoylation. All these factors can potentially vary among cell types and in response to pathophysiological signals. Further studies on such dynamics would be important for better understanding and manipulation of compartmentalized degradation of membrane-related proteins.

Albeit a minority of the entire proteasome pool in cells, MAPs apparently play irreplaceable roles at both cellular and organismal levels, in line with their wide presence at various subcellular locales, in many different cell types, and at different developmental stages. Membrane anchoring of the proteasome is expected to increase its local concentration and/or facilitate substrate capture, as has been demonstrated for other enzymes such as receptor tyrosine kinases (*64*, *65*). The local presence of proteasomes can be particularly convenient for membrane-related events such as ERAD and Gag cleavage, even though these processes have been thought to be controlled solely by cytosolic proteasomes. Consistent with this notion, a recent cryo–electron tomography study visualized 26*S* proteasome clusters on the ER membrane engaging in ERAD (*66*). These proteasomes could well be anchored via Rpt2 myristoylation, which allows for the proteasome to meet the substrates as they exit ER and facilitates rapid degradation on the spot. Although we did not observe grossly elevated ER stress in G2A cells (U2OS and MEF) and liver tissues from the mutant mouse embryos (fig. S5), we would not exclude the possibility that dysregulated ERAD of particular proteins in the absence of MAPs can be severely detrimental under certain conditions and/or in certain cell types that are vulnerable to ER stress.

On the other hand, in light of the exceedingly crowded cellular environment, membranes may serve as a foothold for myristoylated proteasomes and a platform for degradation of nonmembrane-associated proteins, as we also noticed from the proteomic results (Fig. 3D). In vivo, e.g., during neurodevelopment, the membranous compartments of neuronal cells undergo marked remodeling, and local protein synthesis and degradation are tightly coupled in membrane-confined structures such as spines and synapses (*1*, *67*–*70*). This imposes heavy demands on the proteasome system including MAPs, the lack of which can lead to the brain development defects we observed in the G2A/G2A mouse embryos. In addition, cytosolic or nuclear proteins bearing aberrant hydrophobic C termini tend to be unstable and mistargeted to the membrane (*71*, *72*), raising the possibility that they may be cleared by MAPs as a means of protein quality control. Besides misaccumulation of substrates, the elimination of MAPs also indirectly influences protein trafficking and secretion, which could be both cause and consequence of dysregulation of the endomembrane system of the cell. Although the aberrant up-regulation of sorting proteins such as VPS25 and CI-M6PR in G2A MEFs are likely involved, more detailed mechanisms would require further investigation and the exact mediators are probably cell type– and organelle-specific. Some of these changes in G2A cells could, in turn, cause alterations in signal transduction and gene expression (Figs. 3E, 5C, and 6E and fig. S7A). In this sense, compartmentalized protein regulation by MAPs has far-reaching biological significance, as supported by the embryonic lethality and potent tumor-suppressive effect of the Rpt2^G2A/G2A^ mutation. From a therapeutic viewpoint, selective targeting of MAPs while leaving the bulk of cellular proteasomes untouched may be effective in suppressing tumor growth and viral propagation with much-reduced side effects associated with common proteasome inhibitors.

## MATERIALS AND METHODS

### Antibodies and reagents

The rabbit anti–Myr-Rpt2 polyclonal antibody was generated with the peptide antigen (Myr)GQSQSGGHGPGGGKKD-Cys, followed by negative absorption and protein A affinity purification. Information on all the other commercial antibodies and reagents is listed in the Supplementary Materials.

### Plasmids, RNA interference, and gene editing

All cDNAs of human proteasome subunits were originally provided by S. Murata (The University of Tokyo). CD3δ-GFP was a gift from Y. Liu (ShanghaiTech University). hTERT and N-Ras^G12V^ expression constructs were provided by S. Lin and B. Zhao, respectively (Zhejiang University). Rpn11-TBHA, Rpt2-internal Flag (IF), Myr^Src^-Rpt2-G2A, and Myr^Rpt2^-GFPodc have been reported (*27*, *28*). CP8 and uTEV3 were synthesized based on published sequences (*37*, *73*). Other cDNAs were polymerase chain reaction (PCR)–amplified from the ORF LITE human cDNA library (Thermo Fisher Scientific) or reverse-transcribed from WT MEFs. Insertion of short amino acid stretches such as Myr^Src^ (amino acids 1 to 14 of human Src) and the TEV recognition motif was achieved by annealing and ligation of primers containing the corresponding coding sequences. Site-directed mutagenesis was done with QuikChange (Agilent), Gibson Assembly (New England Biolabs), or the related CloneExpress II kit (Vazyme), and all constructs have been fully confirmed by Sanger sequencing.

RNA interference knockdown and simultaneous cDNA re-expression were achieved as reported (*27*) using the pLL3.7 lentiviral vector (from T. Jacks, Massachusetts Institute of Technology) or its modified version, pSuya. For CRISPR-Cas9–mediated Rpt2-G2A knock-in, cells were cotransfected with two guide RNA sequences (in the PX458 vector containing Cas9-2A-GFP, Addgene) targeting intronic sequences flanking exon 2 of the human *PSMC1* gene, a donor plasmid with the G2A mutation and homology arms, and the i53 plasmid for enhancing repair efficiency (*74*). Transfected GFP^+^ cells were enriched by flow cytometry. Single clones were screened and confirmed by genomic DNA sequencing and Western blot analysis. All oligonucleotide sequences are listed in the Supplementary Materials.

### Cell culture, transfection, and infection

WT and G2A MEFs were isolated from E12.5 embryos of the same litter and genotyped. Primary cells were immortalized by infecting with retroviruses expressing SV40 large T/small T antigens (pBabe-T/t) followed by puromycin selection. All other cells were laboratory stocks originally obtained from the American Type Culture Collection, with the exception of SH-SY5Y cells which were purchased from the National Collection of Authenticated Cell Cultures of China. Cells were maintained in Dulbecco’s modified essential medium (DMEM) or RPMI 1640 medium supplemented with 10% fetal bovine serum (FBS) and penicillin/streptomycin (all from Thermo Fisher Scientific). Ciprofloxacin was added periodically to prevent mycoplasma growth. Transfection was done with polyethylenimine or Lipofectamine 2000 according to standard protocols. Retroviral and lentiviral packaging and infection were performed as previously described (*28*).

### Immunoblotting and immunoprecipitation

Samples for routine immunoblotting were prepared in the buffer TBSN [50 mM tris (pH 7.5), 125 mM NaCl, and 0.5 to 1.0% NP-40] supplemented with protease inhibitors (1 mM Pefabloc, 1 mM benzamidine hydrochloride, 1 μM Leupetin, 1 μM E-64, and 1 mM phenylmethanesulfonyl fluoride). When necessary, phosphatase inhibitors were included (10 mM NaF, 20 mM β-glycerolphosphate, 50 nM okadaic acid, and 1 to 10 mM activated orthovanadate). All procedures were performed on ice or at 4°C. For membrane protein extraction, radioimmunoprecipitation assay (RIPA) buffer [20 mM tris-HCl (pH 7.4), 150 mM NaCl, 1% Triton X-100, 0.1% SDS, and 0.5% sodium deoxycholate] or 1% SDS lysis buffer [in 50 mM tris (pH 7.5)] was used. Protein concentration was determined by Bradford protein assay (Bio-Rad) or BCA protein assay (Thermo Fisher Scientific). Samples were mixed with Laemmli sample buffer, boiled at 95°C for 5 to 10 min or incubated at room temperature for 30 to 60 min (for certain membrane proteins), resolved by SDS–polyacrylamide gel electrophoresis (PAGE), and transferred to nitrocellulose membranes for immunoblotting.

To assess sterol-induced degradation of HMGCR (*75*), 8.0 × 10^5^ WT or G2A MEFs were seeded in 6-cm plates. On the third day after seeding, cells were sterol-starved for 16 hours by switching into a cholesterol-depletion medium (DMEM containing 5% lipoprotein-deficient serum, 1 μM lovastatin, and 10 μM mevalonate). Various concentrations of 25-hydroxycholesterol (0/0.1/0.3/1.0 μg/ml) and mevalonate (0/1/3/10 mM) were then added to cells in a cholesterol-depletion medium. After 4 hours of treatment, cells were harvested in RIPA buffer containing protease inhibitors [1 mM phenylmethylsulfonyl fluoride, leupeptin (10 μg/ml), pepstatin A (5 μg/ml), ALLN (25 μg/ml), and 5 μM MG-132]. Cell lysates were centrifuged at 13,000*g* for 10 min, mixed with an equal volume of solubilization buffer [62.5 mM tris-HCl (pH 6.8), 15% SDS, 8 M urea, 10% glycerol, and 100 mM dithiothreitol], and incubated at 37°C for 30 min. Samples were then mixed with Laemmli sample buffer, resolved by SDS-PAGE, and transferred onto a polyvinylidene fluoride membrane for immunoblotting.

For nondenaturing immunoprecipitation, 0.5 to 1.0 mg of cell lysate prepared in TBSN or RIPA buffer was either incubated with 2 to 4 μg of antibody for 1 hour, and then with 8 to 10 μl of protein G agarose (Thermo Fisher Scientific) for another 30 to 45 min, or with 8 to 10 μl anti-Flag resin for 1 hour. For detection of polyubiquitination, cells were lysed under denaturing conditions [50 mM tris (pH 7.5), 125 mM NaCl, 1% SDS supplemented with protease inhibitors, and 20 mM *N*-ethylmaleimide (NEM)]. Concentrated lysates were diluted 10-fold with TBSN buffer with protease inhibitors and NEM, sonicated, and spin-cleared. Supernatants were measured for protein concentration and used for immunoprecipitation. Beads were extensively washed for four times with TBSN buffer and boiled in Laemmli sample buffer for immunoblotting.

### Click chemistry and surface biotinylation

Cells were treated with Alk-Myr (50 μM) for 12 to 24 hours and lysed with TBSN buffer as described above. After streptavidin pull-down (for proteasome isolation) or anti-Flag immunoprecipitation, the beads were washed and incubated in the click reaction solution: 1 mM biotin-PEG3-azide, 150 μM CuSO_4_, 300 μM BTTAA, and 1 mM TCEP (or 5 mM ascorbic acid) at 30°C for 1 hour. The reaction was stopped by adding 1 mM EDTA and boiled for streptavidin–horseradish peroxidase blotting.

For surface biotinylation, cells were washed three times with ice-cold phosphate-buffered saline (PBS) and then labeled with Biotin-LC-Sulfo-NHS (0.5 mg/ml in PBS; Confluore) at 4°C for 1 hour with gentle agitation. The reaction was quenched by three 5-min washes with 100 mM glycine in PBS. Cells were lysed with RIPA buffer and sonicated. Spin-cleared cell lysates were then incubated with streptavidin beads at 4°C for 1 hour. Beads were washed with PBS three times and used for subsequent analysis.

### Proteasome isolation and analyses

Affinity purification of 26*S* proteasomes using the TBHA-streptavidin system and subsequent elution with TEV cleavage for MS analysis were carried out as described (*23*). Measurement of peptidase activity of the proteasome with the fluorogenic substrate Suc-LLVY-AMC, native gel electrophoresis, and sucrose gradient ultracentrifugation was performed as previously reported (*31*, *76*). His-SUMO-Rpt2 purification was done as reported (*27*).

### Electron microscopy

A pre-embedding protocol was used for immunoelectron microscopy (IEM) on brain tissue sections. One-month-old male mice (Rpn11^TBHA/+^) were anesthetized and perfused transcardially with ice-cold PBS (pH 7.4) followed by the fixative [0.1% glutaraldehyde and 4% formaldehyde in 0.1 M phosphate buffer (PB; pH 7.4)]. Mouse brain was quickly dissected and further fixed overnight. Coronal sections (50 μm) were cut using an oscillating microtome (Leica VT1000S) in 0.1 M PB (pH 7.4) and postfixed in the same fixative above for 2 hours. After rinsing with PBS (pH 7.4), the brain sections were incubated with 50 mM glycine in 0.1 M PB for 30 min and then permeabilized with 0.01% Triton X-100 in 0.1 M PB for 15 min. After blocking with 0.1% BSA-c (Aurion) in 0.1 M PB, the sections were incubated with a rabbit monoclonal anti-HA antibody (1:100; Cell Signaling Technology, #3724) at 4°C overnight. Following extensive washes, the secondary antibody (1:100; NANOGOLD Goat anti-rabbit IgG, Nanoprobes Inc.) was added. After overnight incubation at 4°C and washing in 0.1 M PB (pH 7.4), the brain sections were again postfixed with 2.5% glutaraldehyde in 0.1 M PB and treated with the silver enhancement kit (Nanoprobes Inc.) according to the manufacturer’s procedure. Samples were post-stained in 1% OsO_4_ and 2% uranyl acetate, dehydrated, and embedded in epon, and ultrathin sections were cut for imaging.

For IEM on MEFs, a post-embedding method was used. Cells grown on sapphire discs were cryo-immobilized by high-pressure freezing (Wohlwend HPF COMPACT 01) and freeze-substituted in 0.1% uranyl acetate and 5% H_2_O in acetone, and then infiltrated and embedded in LRW resin or HM20 resin according to the instructions. Ultrathin sections (90 nm thick) were mounted on nickel grids coated with formvar film for immunogold staining. Anti-HA (BioLegend, HA.11) or anti-PSMA2 (Cell Signaling Technology, #2455) antibodies were used at 1:10 or 1:20 dilutions, and goat anti-mouse (G7652) or goat anti-rabbit (G7402) secondary antibodies from Sigma-Aldrich were diluted 1:50. Grids with sections were incubated in solutions by floating the section side down on a drop of solution and transferred sequentially from drop to complete the immunostaining. To cover a grid, 5- to 50-μl drops of the solution were placed on a piece of parafilm in a petri dish kept in a moist chamber to avoid drying of the solution. A gold enhancement kit, GoldEnhance-EM plus 2114 (Nanoprobes Inc.), was used to enlarge the labeled colloidal gold for 3 to 10 min. After rinsing in distilled water, the section was post-stained with 3% uranyl acetate and Sato’s lead.

For morphology analysis, cells grown on sapphire discs were cryo-immobilized as above with 0.25-mm-deep HPF carriers as caps and 1-hexadecene as a cryoprotectant. For optimal ultrastructural preservation, samples were transferred to a frozen freeze-substitution medium (acetone containing 1% OsO_4_, 0.1% uranyl acetate, and 5% H_2_O) under liquid nitrogen and placed in an automatic freeze-substitution system (Leica AFS2, Leica Microsystems) precooled to −90°C. Freeze-substitution was carried out at −90°C for 8 hours. The samples were subsequently warmed to −60°C over 3 hours, kept at −60°C for 3 hours, and warmed to −30°C over 3 hours, kept at −30°C for 3 hours. Then, the samples were warmed to 4°C over 3 hours and kept at 4°C for 15 min before washing with anhydrous acetone. Samples were then infiltrated and embedded in SPI-Pon 812 resin. Ninety-nanometer-thick ultrathin sections were cut with an ultramicrotome (Leica EM UC7, Leica Microsystems) and stained with 3% uranyl acetate in 70% methanol/H_2_O for 7 min, followed by Sato’s lead for 2 min.

All EM images were acquired on a TECNAI G2 Spirit transmission electron microscope (FEI; Eindhoven, Netherlands) operated at 120 kV. Images with clear membrane/organelle structures were chosen for further analysis.

### Fluorescence microscopy and immunohistochemistry

Immunofluorescence staining of cells was performed as described in (*77*). Images were taken with a spinning disk confocal microscope (Andor) or laser scanning microscope (ZEISS LSM 880 with AiryScan) under a 60× or 100× oil lens. LysoTracker labeling was done as instructed by the manufacturer, and live cells were imaged with a DV ELITE microscope (Applied Precision Instruments) under a 60× oil lens. Bright-field images were captured using a Nikon Eclipse TS100 inverted microscope with an Oplenic LCC60-HD camera.

For TIRF, U2OS (parental and G2A knock-in) cells were transfected with mG [membrane-targeted GFP (*78*)] or Rpn10-GFP. Fixed cells were imaged with an Olympus IX83 microscope using a 100×/1.5 NA oil objective under the TIRF-SIM mode. Penetration depth was set at 200 nm. SIM images were acquired and reconstructed using the Wiener deconvolution algorithm as previously described (*79*, *80*). Regular wide-field images were also taken to demonstrate the pancellular distribution of the bulk of Rpn10-GFP.

Immunohistochemistry staining of frozen sections of the embryonic brain was performed as previously described (*81*). Anti-CD31 and hematoxylin and eosin staining of paraffin-embedded embryonic tissues was done according to standard protocols.

### LysoTracker staining for flow cytometry

MEFs were first infected with lentiviruses generated from the pSuya-GFP backbone expressing individual shRNAs. Three days after infection, cell populations were stained with LysoTracker Red (1:10,000, 37°C for 15 min). Cells were trypsinized, resuspended in PBS containing 2% FBS, passed through a cell strainer mesh, and analyzed on a CytoFLEX S flow cytometer (Beckman Coulter). Uninfected cells and unstained cells were used as negative controls for gating GFP^+^ and LysoTracker^+^ cells, respectively. For each sample, the integrated LysoTracker fluorescence intensity of all GFP^+^ cells was calculated by the FlowJo software.

### Cell and tissue fractionation

Two 10-cm plates of cells grown at near confluency were scraped off, pelleted, and washed with PBS. Each cell pellet was resuspended in a hypotonic lysis buffer [5 mM Hepes (pH 7.9), 5 mM MgCl_2_, supplemented with 1 mM adenosine 5′-triphosphate, and protease inhibitors]. Digitonin was also included at a final concentration of 0.005% (w/v) to facilitate cell lysis and centrifugal removal of nuclei. After being swollen on ice for 5 min, cells were broken by passing through a 23G syringe needle 20 times. A small aliquote of the sample was boiled in SDS loading buffer as the whole-cell lysate, while the remainder was centrifuged at 1400*g* at 4°C for 20 min to remove unbroken cells, nuclei, and large cytoskeleton complexes (which all contain proteasomes). The resulting supernatant was further centrifuged at 10,000*g* at 4°C for 10 min. The soluble (cytosolic) fraction was transferred to a new tube, and the membrane pellet was washed twice with the same hypotonic buffer as above. RIPA buffer was added to dissolve membrane proteins from the pellet. After centrifugation at 21,130*g* at 4°C for 10 min, the supernatant was boiled as the membrane fraction.

For the isolation of membranes from mouse tissues, different organs were disrupted with a glass/Teflon homogenizer in Hepes buffer [20 mM Hepes (pH 7.4), 0.25 M sucrose, 1 mM EDTA, and 1 mM EGTA]. Tissue homogenates were cleared twice by centrifugation at 1000*g*. The supernatants were then centrifuged at 15,000*g*, and the resulting supernatants were further spun at 198,000*g*. All centrifugation steps were performed at 4°C for 10 min. The final membrane pellets containing mostly plasma membrane, microsomal membrane, Golgi, and endosomes were dissolved in RIPA buffer or 1×Laemmli sample buffer for Western blot analysis.

### Mice

Rpn11-TBHA knock-in mice (*Psmd14^TBHA^*) generated at the Transgenic Core of University of California - San Diego have been reported (*31*). Rpt2-G2A knock-in mice (*Psmc1^G2A^*) were created by CRISPR-Cas9–mediated gene editing at Biocytogen (Beijing, China). All genomic modifications have been confirmed by Southern blot, PCR genotyping, and Sanger sequencing. Both strains are on the C57Bl/6 background and have been extensively and periodically back-crossed with WT mice. Immunocompromized mice (Nu/Nu) were purchased from Shanghai SLAC Laboratory Animal Co. Ltd. (Shanghai, China). All animals were housed at the Laboratory Animal Center at Zhejiang University, and all routine husbandry and tumor xenograft studies were in full compliance with the policies of the Institutional Animal Care and Use Committee and approved animal protocols (#11828 and #22470).

For the tumor xenograft study, 3.0 × 10^6^ oncogene-transformed MEF cells were mixed 1:1 (v/v) with Matrigel and injected subcutaneously into the flank of each nude mouse (5-week-old females). At the beginning of the experiment, all nude mice (purchased from the same source at the same time) were randomly assigned to three groups, nine mice each, according to the standard protocols used in the literature. Tumor volume was monitored regularly with a digital caliper (not blinded to the researcher) and was calculated by the following formula: *V* = 1/2 × width^2^ × length. No animals were excluded.

### Cell adhesion, migration, and viability assays

For cell adhesion assays, purified ECM components were prepared in PBS according to the manufacturer’s instruction and used to coat 12-well plates as follows: fibronectin (1 μg/cm^2^), collagen type IV (0.1 mg/cm^2^), laminin (1 μg/cm^2^), and vitronectin (0.1 μg/cm^2^). Coated plates were air-dried at 37°C overnight. Cells were dissociated with Versene and resuspended in a serum-free medium. After cell counting, 2.0 × 10^5^ cells were added to each coated well and allowed to attach at 37°C for 30 min. Unbound cells were removed by washing twice with PBS, and the remaining cells were fixed with 4% paraformaldehyde and stained with 0.5% crystal violet. After extensive washing with distilled water, air-dried plates were photographed. For each condition, a total of 18 random eye fields from triplicate wells were imaged, and the numbers of adhered cells were quantified by ImageJ. Alternatively, the crystal violet of the stained cells was dissolved with 200 μl of ethanol plus 1% (v/v) of concentrated HCl, and Abs590 was measured on a Tecan multiwell plate reader.

For wound-healing (scratch) assays, 8.0 × 10^5^ MEFs were seeded in a 12-well plate for 24 hours. A wound through the confluent cell monolayer was made with a P200 micropipette tip. Cells were washed three times with PBS and fresh medium was added. Photos of the wound areas (time “0”) were taken. Cells were allowed to migrate for 18 hours, and the same eye fields were imaged again. Reduction in the wound area was measured by ImageJ and used to reflect cell migration.

For transwell assays, 2.0 × 10^4^ MEFs resuspended in serum-free medium were added to the top of a Boyden chamber, while the bottom chamber was filled with DMEM + 10% FBS. Cells were incubated at 37°C for 24 hours. Migrated cells on the porous membrane were fix-stained with crystal violet as above, imaged, and counted. Cell proliferation and viability were measured by the CellTiter 96 AQueous One Solution Cell Proliferation Assay (Promega) or using the CCK-8 kit as previously reported (*31*).

### Quantitative proteomics

Immortalized MEFs were grown in light or heavy (^13^C_6_-lysine/^13^C_6_^15^N_4_-arginine) SILAC medium for more than seven passages. The cells were washed twice with PBS, harvested in a denaturing lysis buffer [8 M urea and 100 mM tris (pH 8.5)], and sonicated at 4°C for 10 min. Samples were reduced with 5 mM TCEP (Sigma-Aldrich) at room temperature for 20 min and alkylated with 10 mM iodoacetamide (Sigma-Aldrich) for 15 min in the dark. Urea concentration was diluted to 2 M with 100 mM tris (pH 8.5) before trypsin (Promega) was added at a 100:1 protein:enzyme ratio. After digestion at 37°C for 16 hours, the peptides were loaded onto a column which was filled with C18 (3 μm) and SCX (5 μm, Phenomenex) resins and eluted with increasing concentrations of ammonium acetate (25 mM, 75 mM, 100 mM, 150 mM, 200 mM, 275 mM, 375 mM, 500 mM, and 1 M). Fractions were vacuum-dried and resuspended in water with 0.1% formic acid. MS experiments were performed on a Q Exactive HF-X instrument (Thermo Fisher Scientific) coupled with an Easy-nLC 1200 liquid chromatography system. Mobile phase A was water, and mobile phase B was 80% acetonitrile, both containing 0.1% formic acid. Samples were loaded directly onto a C18 reverse phase column (75 μm × 15 cm, 1.9 μm C18). Peptides were separated using a linear gradient of 8 to 40% B for 50 min, 40 to 100% B for 5 min, and 100% B for 5 min. Data-dependent analysis was performed by acquiring a full scan over a mass/charge ratio (*m/z*) range of 350 to 1500 in the Orbitrap at R = 60,000, NCE = 27, with a normalized automatic gain control (AGC) target of 3 × 10^6^. The AGC targets and maximum ion injection time for the MS2 scans were 5 × 10^4^ and 30 ms, respectively. Precursors of the +1, +8, or above, or unassigned charge states were rejected; exclusion of isotopes was disabled; dynamic exclusion was set to 40 s. Raw data were searched with the MaxQuant software (version 1.6.10.43) against the mouse database downloaded from UniProt. Peptides were labeled as Arg^10^ and Lys^6^; the fixed modification was carbamidomethyl; the main search peptide tolerance was 4.5 parts per million.

### Transcriptomic analysis

Total RNA was isolated from immortalized MEFs or three pairs (WT and G2A littermates) of E12.5 fetal livers using the TRIzol method. mRNA sequencing was done by BGI (Shenzhen, China). Raw reads were trimmed to 50 base pairs and mapped to the mouse genome (mm9) using Tophat v2.1.1 with default parameters. Only uniquely mapped reads were subsequently assembled into transcripts guided by the reference annotation (UCSC gene models) using Cufflinks v2.2.1. The RNA abundance of each gene was quantified with FPKM (fragments per kilobase of exon per million mapped fragments). Genes with FPKM < 1 in all samples were excluded, and for the remaining genes, all FPKM values smaller than 1 were set to 1 in subsequent analyses. Differentially expressed genes were identified using the criteria |FC| > 2. Gene Ontology enrichment was performed using the “Metascape” web server.

### Data analyses

Unless otherwise noted, all Western blots were repeated at least three times (biological repeats) by independent laboratory members and quantified by ImageJ (https://imagej.nih.gov/ij/). Fluorescence microscopy images were processed using the MetaMorph software package (Molecular Devices) or ImageJ. Sequence alignment was done by ClustalW (www.genome.jp/tools-bin/clustalw) and ESPript (http://espript.ibcp.fr/ESPript/cgi-bin/ESPript.cgi). Statistical analyses were performed with GraphPad Prism, and quantitative results are shown as means ± SD. No data were excluded.
