# Supplementary material for: Lipid-anchored proteasomes control membrane protein homeostasis
Source: Sci Adv. 2023 Nov 29;9(48):eadj4605. doi: 10.1126/sciadv.adj4605 (PMC10686573; doi:10.1126/sciadv.adj4605)
Supplement: Supplementary file 1 — Supplementary Materials and Methods Figs. S1 to S8 Legends for tables S1 to S4 [file sciadv.adj4605_sm.pdf]

Science Advances
AAAS

Supplementary Materials for

Lipid-anchored proteasomes control membrane protein homeostasis

Ruizhu Zhang *et al.*

Corresponding author: Xing Guo, xguo@zju.edu.cn; Bing Yang, bingyang@zju.edu.cn

Sci. Adv. **9**, eadj4605 (2023)
DOI: 10.1126/sciadv.adj4605

The PDF file includes:

Supplementary Materials and Methods
Figs. S1 to S8
Legends for tables S1 to S4

Other Supplementary Material for this manuscript includes the following:

Tables S1 to S4

Supplementary Materials and Methods

Antibody information

Antibodies	Source	Catalog No. and RRID	Application
20S α123567	Enzo Life Sciences	Cat# BML-PW8195; RRID: AB_11177877	1:5,000 (WB)
AKT1	HUABIO	Cat# EM40507	1:1,000 (WB)
AKT1 (pS473)	HUABIO	Cat# ET1607-73	1:1,000 (WB)
ATF4	HUABIO	Cat# ET1612-37	1:1,000 (WB)
BIP	HUABIO	Cat# M1506-2	1:1,000 (WB)
Calnexin	Cell Signaling	Cat# 2679T; RRID: AB_2228381	1:400 (ICC)
Calreticulin	HUABIO	Cat# ET1608-60	1:1000 (ICC)
CD31	Cell Signaling	Cat# 77699S; RRID: AB_2722705	1:200 (IHC)
CHOP	HUABIO	Cat# ET1703-05	1:1,000 (WB)
CI-M6PR	HUABIO	Cat# ET1602-5	1:2,000 (WB) 1:300 (ICC)
Clathrin Heavy Chain (CHC)	BD Biosciences	Cat# 610500; RRID: AB_397866	1:1,000 (WB)
Cyclin D1	HUABIO	Cat# ET1601-31	1:1,000 (WB)
E-cadherin	Affinity Biosciences	Cat# BF0219; RRID: AB_2833860	1:1,000 (WB)
EGFR	Cell Signaling	Cat# 4267T; RRID: AB_2799342	1:1,000 (WB) 1:250 (ICC)
EGFR (Tyr1068)	Cell Signaling	Cat# 3777T; RRID: AB_1903957	1:1,000 (WB)
Erk (pY204)	Santa Cruz Biotechnology	Cat# sc-7383; RRID: AB_627545	1:500 (WB)
Erk1/2	HUABIO	Cat# ET1601-29	1:1,000 (WB)
FAK	Proteintech	Cat# 66258-1-Ig; RRID: AB_2881646	1: 200 (IF)
Flag-HRP	Shanghai Genomics Technology	Cat# GNI4310-FG; RRID: AB_2885081	1:5,000 (WB)
Flag-Tag	AbMart	Cat# M20008S; RRID: AB_2713960	1:5,000 (WB)
GAG	HUABIO	Cat# ER50102	1:1,000 (WB)
GAPDH	Millipore	Cat# CB1001; RRID: AB_2107426	1:1,000 (WB)
GFP	AbMart	Cat# M20004L; RRID: AB_2619674	1:1,000 (WB)
GOLGA4	ABclonal	Cat# A10216; RRID: AB_2757739	1:1,000 (WB)

HA-Tag	Cell Signaling	Cat# 3724S; RRID: AB_1549585	1:5,000 (WB)
HA-Tag (HA.11)	BioLegend	Cat# 901501; RRID: AB_2565006	1:1,000 (WB) 1:10-20 (IEM)
HMGCR	Produced from hybridoma (Baoliang Song)	IgG-A9	1:1,000 (WB)
Hsp90α	HUABIO	Cat# M1603-3	1:5,000 (WB)
ICAM1	HUABIO	Cat# ET1609-46	1:1,000 (WB)
ITGA2/Integrin α2	HUABIO	Cat# ET1611-57	1:1,000 (WB)
ITGA3/Integrin α3	HUABIO	Cat# HA500111; RRID: AB_11156484	1:1,000 (WB)
ITGAV/Integrin αV	HUABIO	Cat# ET1610-15	1:1,000 (WB) 1:300 (ICC)
Kif3A	HUABIO	Cat# ER1803-40	1:1,000 (WB)
Lamin A/C	Cell Signaling	Cat# 2032; RRID: AB_2136278	1:1,000 (WB)
LAMP1	Cell Signaling	Cat# 9091T; RRID: AB_2687579	1:1,000 (WB)
mCherry	HUABIO	Cat# HA500049	1:1,000 (WB)
NPC1/Niemann Pick C1	HUABIO	Cat# ET7107-57	1:1,000 (WB)
Nrf1/NFE2L1	Proteintech	Cat# 12936-1-AP; RRID: AB_2267298	1:1,000 (WB) 1:200 (IP)
PAX6	Medical & Biological laboratories	Cat# PD022; RRID: AB_ 1520876	1:1,000 (IHC)
PCNA	Santa Cruz Biotechnology	Cat# SC-56; RRID: AB_628110	1:1,000 (WB)
PDGFRα	Cell Signaling	Cat# 3174S; RRID: AB_2162345	1:1,000 (WB) 1:200 (IP)
PD-L1	Abcam	Cat# Ab282458	1:1,000 (WB)
Piezo1	Proteintech	Cat# 15939-1-AP; RRID: AB_2231460	1:500 (WB)
Profilin-2	Santa Cruz Biotechnology	Cat# 100955; RRID: AB_2163221	1:1,000 (WB)
RKIP	Cell Signaling	Cat# 13006S; RRID: AB_2798085	1:1,000 (WB)
Rpn1/PSMD2	Bethyl Laboratories	Cat# A303-854A; RRID: AB_2620205	1:1,000 (WB)
Rpn10/S5a/PSMD4	Cell Signaling	Cat# 12441S; RRID: AB_2797916	1:1,000 (WB)
Rpn11/PSMD14	Cell Signaling	Cat# 4197; RRID: AB_11178935	1:1,000 (WB)
Rpn2/PSMD1	Santa Cruz Biotechnology	Cat# sc-166038	1:1,000 (WB)

Rpt2/PSMC1	Proteintech	Cat# 11196-1-AP; RRID: AB_2284521	1:1,000 (WB)
Rpt3/PSMC4	Bethyl Laboratories	Cat# A303-849A; RRID: AB_2620200	1:1,000 (WB)
Rpt6/PSMC5	Enzo Life Sciences	Cat# BML-PW-9265-0100; RRID: AB_10541436	1:1,000 (WB)
RRas	HUABIO	Cat# ER60170	1:1,000 (WB)
SOX2	Abcam	Cat# Ab79351; RRID: AB_10710406	1:200 (IHC)
Src	Cell Signaling	Cat# 2109S; RRID: AB_2106059	1:1,000 (WB)
STIM1	Abcam	Cat# Ab108994; RRID: AB_10859115	1:1,000 (WB)
TC-PTP/PTPN2	R&D Systems	Cat# MAB1930; RRID: AB_2173232	1:1,000 (WB)
TGF-β Receptor III	Cell Signaling	Cat# 5544S; RRID: AB_10698740	1:1,000 (WB)
PolyUb (K48-linked)	Cell Signaling	Cat# 8081S; RRID: AB_10859893	1:1000 (WB)
Vinculin	HUABIO	Cat# ET1705-94	1:1000 (WB)
VPS25	Proteintech	Cat# 15669-1-AP; RRID: AB_2215019	1:1,000 (WB) 1:200 (IP)
ZO2	HUABIO	Cat# R1402-2	1:1,000 (WB) 1:200 (IP)
α2/PSMA2	Cell Signaling	Cat# 2455S	1:10-20 (IEM)
α5/PSMA5	Cell Signaling	Cat# 2457S; RRID: AB_823611	1:1,000 (WB)
α7/PSMA3	Cell Signaling	Cat# 12446S; RRID: AB_2797918	1:1,000 (WB)
β5/PSMB5	Cell Signaling	Cat# 12919S; RRID: AB_ 2798061	1:1,000 (WB)
β-tubulin	Cell Signaling	Cat# 15115; RRID: AB_2798712	1:1,000 (WB)
γ-tubulin	HUABIO	Cat# M1701-13	1:1,000 (WB)
Peroxidase Streptavidin	Jackson Immuno Research	Cat#016-030-084; RRID: AB_2337238	1:5,000 (WB)
Peroxidase AffiniPure Goat Anti-Rabbit IgG(H+L)	Jackson Immuno Research	Cat# 111-035-003; RRID: AB_2313567	1:10,000 (WB)
Peroxidase AffiniPure Goat Anti-Mouse IgG(H+L)	Jackson Immuno Research	Cat#115-035-003; RRID: AB_10015289	1:10,000 (WB)
Peroxidase AffiniPure Goat Anti-Mouse IgG, Light Chain Specific	Jackson Immuno Research	Cat# 115-035-174; RRID: AB_2338512	1:10,000 (WB)
Peroxidase Monoclonal Mouse Anti-Rabbit IgG, Light Chain Specific	Jackson Immuno Research	Cat# 211-032-171; RRID: AB_2339149	1:10,000 (WB)

Alexa 568-goat anti-mouse secondary antibody	Thermo Fisher	Cat# 1841757; RRID: AB_144696	1:5,000 (ICC)
Alexa 488-goat anti-rabbit secondary antibody	Thermo Fisher	Cat# 1851447; RRID: AB_2576217	1:5,000 (ICC)
Alexa Fluor™ 568 goat anti-rabbit IgG (H+L)	Thermo Fisher	Cat# 1832035; RRID: AB_10563566	1:5,000 (ICC)
Alexa Fluor™ 488 goat anti-mouse IgG (H+L)	Thermo Fisher	Cat# 1874804; RRID: AB_2534088	1:5,000 (ICC)

Reagent information

**Chemicals and Reagents**	**Source**	**Catalog No.**
Alkynyl Myristic Acid	Click Chemistry Tools	Cat#1164-5
Biotin-LC-Sulfo-NHS	Confluore	Cat# BBBA-8; CAS# 191671-46-2
Biotin-PEG3-Azide	Click Chemistry Tools	Cat# AZ 104-25
Bortezomib	ApexBio	Cat# A2614
BTTAA	Click Chemistry Tools	Cat# 1236-100
Cell Counting Kit-8	Beyotime	Cat# C0039
Ciprofloxacin	Sigma	Cat# 17850
Collagen Type IV from human placenta	Sigma-Aldrich	Cat# C5533
Cycloheximide	Sigma	Cat# C7698
Digitonin	Sigma	Cat# D141-100MG
Fibronectin from bovine plasma	Sigma-Aldrich	Cat# F4759
HisPurTM Ni-NTA Resin	Thermo Fisher	Cat# 25214
Human EGF	Peprotech	Cat# Q99075
Hygromycin B	Sigma	Cat# V900372
IMP-1088	Cayman Chemical	Cat# HY-112258
IPTG	Amresco	Cat# C0039
Laminin from human placenta	Sigma-Aldrich	Cat# L6274
L-ascorbic acid	Sigma	Cat# A7506
L-Arginine:HCl (13C6; 15N4)	Cambridge Isotope Laboratories, Inc.	Cat# CNLM-539-H-0.1; CAS# 202468-25-5
Lipofectamine™2000 Transfection Regent	Thermo Fisher	Cat# 11668500

L-Lysine:2HCl (13C6)	Cambridge Isotope Laboratories, Inc.	Cat# CLM-2247-H-0.25; CAS# 201740-81-0
Lyso-Tracker (Red)	Beyotime	Cat# C1046
Matrigel	Corning	Cat# 354234
Medium for SILAC	Thermo Fisher	Cat# 88368
Monensin	SelleckChem	Cat# s2324
MG-132	SelleckChem	Cat# S2619; CAS# 1211877-36-9
Pierce™ High Capacity Streptavidin Agarose Resin	Thermo Fisher	Cat# 20357
Pierce™ Protein G Agarose	Thermo Fisher	Cat# 20397
Polybrene	Sigma	Cat# TR-1003
Polyethylenimine（PEI）	Polysciences	Cat# 23966-1
Puromycin	Thermo Fisher	Cat# A111380
SYBR@ Premix Ex Taq™ II	Takara	Cat# 639676
Trypsin/EDTA	Yeasen	Cat# 40126ES60
Tunicamycin	ApexBio	Cat# B7417
Versene	Thermo Fisher	Cat# 15040066
Vitronectin from human plasma	Sigma-Aldrich	Cat# V8379

PCR Primers

pQCXIP-Rpt2-WT-F	AGCACCGGTACCATGGGTCAAAGTCAGAGTGGTGG
pQCXIP-Rpt2-G2A-F	AGCACCGGTACCATGGCTCAAAGTCAGAGTGGTGG
pQCXIP-Rpt2-WT-R	AGCCTCGAGTTAGAGATACAGCCCCTCAGGGGTGCC
His-SUMO-hRpt2-F	CACAGAGAACAGATTGGTGGAGGTCAAAGTCAGAGTGGTGGTC
His-SUMO-hRpt2-R	GAGTGCGGCCGCAAGCTTGTCGACTTAGAGATACAGCCCCTC
mLYOX-F	CGGCCGCACCGGTCCTCGAGACCATGCGTTTCGCCTGGGCTG
mLYOX-R	ATCCGTTAATTAAGCAATTGGCATACGGTGAAATTGTGCAGCCTG
mILEUA-F	GCACCGGTCCTCGAGACCATGGAGCAGCTGAGTTCAGCC
mILEUA-R	GCAATTGGCTGGGGAACAAACCCTG
mTIMP3-F	CCTCGAGACCATGACTCCCTGGCTTGGGCTT
mTIMP3-R	GCAATTGGCGGGGTCTGTGGCGTT
hTERT-qPCR-F	GAGCTGCTCAGGTCTTTCTTT
hTERT-qPCR-R	CCTCTTCAAGTGCTGTCTGATT
mGOLGA4-qPCR-F	TCCTTAATTGTGTCTCAGCCC
mGOLGA4-qPCR-R	CAGTTCTTTCACCCTTTGCTC

muXBP1-qPCR-F	CAGACTACGTGCACCTCTGC
muXBP1-qPCR-R	CAGGGTCCAACTTGTCCAGAAT
huXBP1-qPCR-F	CAGACTACGTGCACCTCTGC
huXBP1-qPCR-R	CTGGGTCCAAGTTGTCCAGAAT
msXBP1-qPCR-F	GCTGAGTCCGCAGCAGGT
msXBP1-qPCR-R	CAGGGTCCAACTTGTCCAGAAT
hsXBP1-qPCR-F	GCTGAGTCCGCAGCAGGT
hsXBP1-qPCR-R	CTGGGTCCAAGTTGTCCAGAAT
mtXBP1-qPCR-F	TGAAAAACAGAGTAGCAGCGCAGA
mtXBP1-qPCR-R	CCCAAGCGTGTTCTTAACTC
htXBP1-qPCR-F	TGAAAAACAGAGTAGCAGCTCAGA
htXBP1-qPCR-R	CCCAAGCGCTGTCTTAACTC
GAPDH-qPCR-F	ACCATCTTCCAGGAGCGAGA
GAPDH-qPCR-R	GGGCCATCCACAGTCTTCTG
mBip-qPCR-F	CATGGTTCTCACTAAAATGAAGG
mBip-qPCR-R	GCTGGTACAGTAACAACTG
mCHOP-qPCR-F	CTGCCTTTCACCTTGGAGAC
mCHOP-qPCR-R	CGTTTCCTGGGGATGAGATA

Other oligonucleotide sequences

hRpt2-gRNA1-F	CACCGTAAGATCACCCCCCTCTTGA
hRpt2-gRNA1-R	AAACTCAAGAGGGGGGTGATCTTAC
hRpt2-gRNA2-F	CACCGTCTCGGCTGAGAAGTTCTG
hRpt2-gRNA2-R	AAACCAGAACTTCTCAGCCGAGAC
shRNA-hRpt2	GGGGAGTTGCCCAGAGGAA
shRNA-mRpt2	GGAAAATGGTTGGGAGATT
shRNA-mM6PR#1	GCATGGTGTCAGAAGACAA
shRNA-mM6PR#2	GGTCCTTGATGCACACTTA
shRNA-mVPS25#1	CCACAGTGCCACGGAGAAA
shRNA-mGOGA4#1	GGTTGACAGTGTTCAGAAA
shRNA-mGOGA4#2	GGTTGTTCTCTGAGTGAAA
shRNA-mGOGA4#3	GAATGTTGATGCTCCCAAA
TEV-AgeI-F	CCGGCGAGAATCTGTACTTTCAAGGAC
TEV-AgeI-R	CCGGGTCCTTGAAAGTACAGATTCTCG
Myr^{Src}-NheI-F	CTAGCACCATGGGGAGCAGCAAGAGCAAGCCCAAGGATCCCAGCCA GCGCA
Myr^{Src}-AgeI-R	CCGGTGCGCTGGCTGGGATCCTTGGGCTTGCTCTTGCTGCTCCCCA TGGTG

hRpt2-G2A donor sequence:

aagaaatggaaacacagtaagtggctgtgctggaacacaagccccatcgttttttttctagaacagtagagaaacacccaggttttggcattcagca aacctgaatgtgattcctggctccatctctgacaagctttgtgacaacaggcaagtcatttaagctctctgggccttggtcttcttatctgcaaatgagggg gtaatgatggtaccagtgtcatcgtgtttgtaagaattaagtgggataatacttggagaatgtttaggacactgcctggcatatagtaggggttcagtaat tgatgtacttgttatttaagactgagaaacatgtaaaatgcccaagaggtgaagaagtgttggtgtattaatttagaagtgcctgtgtggagctgatttga gggagctgagtgggcccttctaatcagtaagttcactcatctttatatttgaaatgccaccatttctgagacattgctcagtgttaagcattgtcctataact gtagggtctcttaaaatcaggaatcccactgagtttattaatacactaagttcttaacaccttaaggtgatggagcttttcagattgagaagaaagacct

gccccagagccccctggctttaggggtaggaattggataaggtgctattggaaggaaagtcttctggatcttggtgtgatctaagtaaggggtaaatg
attggtaaacgatttttgagtgtgtaaaatgacgtagctttcatagcctgattttatggaatacattcttgatagcttcagtccggtagtttcctatcaagagat
*gtgaagctccatattttcacc*ggt**tcaagaggggggtgatctta**agactctcagcctctttagttgtatctacttctcaatagagttgttcttgattctaaga
tgcaatcaattgccaggtgcaccagtaatctatcgaacatcttttcagggaaaaagaaattctgccacactaaatgcacacattgactgcaagtatta
catcttgggttcagaaatgttcaaatgagggagaaatgttactcacaggacatgttatagactcacaggacagtataaagttattcttttgaagtgatcat
attctgaattcatctgtgcctgatttttctcctccag**GCTCAAAGTCAGAGTGGTGGCCATGGTCCTGGAGGTGGCAAGA**
AGGATGACAAGgtaaatatgccagatttgtcctgtgatatgagcaaattgtggaaaatgtcatgg**gtctcggctgagaagttctg**tac*agatt*
catgaattttaaaaagtagaatgatccagtagtttttaaactagcctgtcctacagatacaataaatatgcttttcatgctttagaaaaggttattgacctaa
ccatcagataggtatttatgagttatttatttgtgtaaaagcaatgacattatagaagtattaaagtaaattgcctataattttggtgtctaatagcctgaggt
atgaagattgtctcaggttacctaaacttttggcatataacttgattcttaaccctctataccttattggaataatatggctaattgtcacttatgtgtatggtaa
aacgattacatttattttcatttgttattattattattattgagacggagtcacccaggctggagtgcagtggcatgatctcggctcactgcaacctccgcctc
ctgggttcaagcaattctcctgtctcagcctcccgagtagctgggattacaggtgcctgccaccacacccggctaatttttgtatttttagtagagatgga
gtttcaccatgttggccaagctggtctcaaactcctgacctcaagtgatcctcccgcctcagcctcccaaaatgctgggattacatacgtgagccacca
cacccagcctacatttacttattataatcatatttacttattaaaattgcaggaatgtgaaacaagtcaggtttttaataacatcctaaaagataaggaaa
attgtaccacctgaaaatagatgatggtggtacagaaataaatgtgtgttttaatatgattttcatgtgattttttttcctgctattctaacaactcaaggaca
agaaaaagaaatatgaacctcctgtaccaactagagtggggaaaaagaagaagaaaacaaagggaccagatgctgccagcaaactgccact
gggtaatgacatggcttctccttgccatctttccagttcttaggataaaccatctctgtgcatgtagcttagagtctgaaaggggtaacaggaagtagag
gggcaactgaagctctagctttgtgattcatttctacctcactcttgagtgtggcattcctctaggttggcatacaagtta

aag…acc: LHA (Left homology arm)
aga…tta: RHA (Right homology arm)
ggt**tcaagaggggggtgatctta**: sgRNA#1 with mutated PAM (reverse)
gtctcggctgagaagttctgtac: sgRNA#2 with mutated PAM
GCT…AAG: Exon2
GCT: G2A mutation

Supplementary Figures

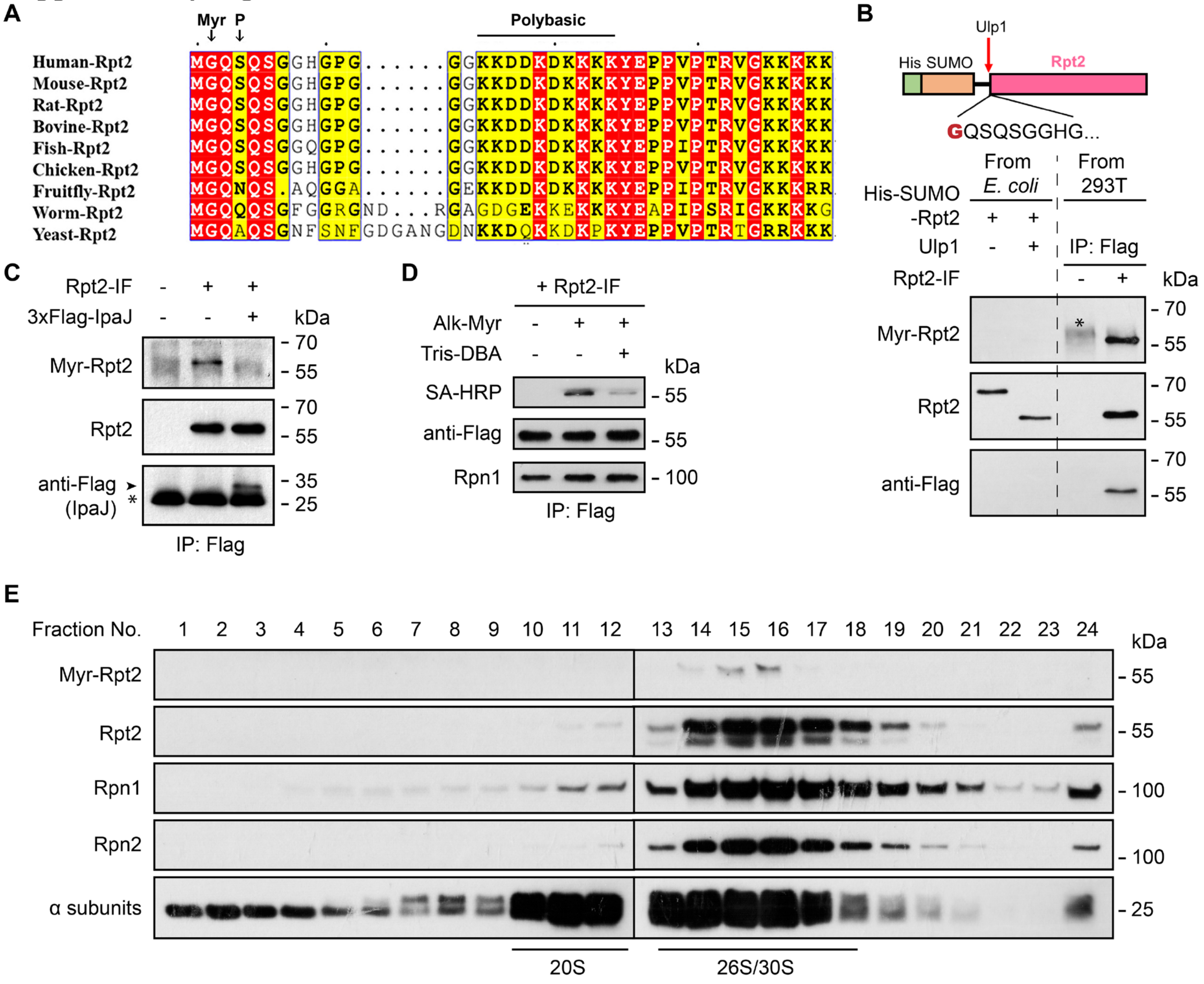

fig. S1. Detection of Rpt2 N-myristoylation.

(**A**) Alignment of Rpt2 N-terminal sequences from different species. The N-myristoylation site (Gly2), phosphosite Ser4 and the nearby polybasic stretch are indicated.

(**B**) Validation of the Rpt2 myristoylation-specific antibody. (Top) A schematic of recombinant His-SUMO-Rpt2 purified from E. coli. After Ulp1 cleavage, the His-SUMO tag was removed, leaving a Rpt2 protein with an unmodified N-termimus. (Bottom) Purified His-SUMO-Rpt2 proteins before and after Ulp1 cleavage were probed with the indicated antibodies. WT Rpt2-IF expressed in 293T cells was used as a positive control for Rpt2 myristoylation. Asterisk, a non-specific band.

(**C**) 293T cells were transfected with Rpt2-IF and IpaJ-3XFlag as indicated. Cells were lysed 24 h later for anti-Flag immunoprecipitation and immunoblotting. Arrowhead, Flag-tagged IpaJ. Asterisk, the light chain of the anti-Flag antibody.

(**D**) 293T cells were transfected with Rpt2-IF (WT) and immediately treated with Alk-Myr in the absence or presence of the NMT1 inhibitor, Tris-DBA (10 μg/ml), as indicated. Rpt2-IF was immunoprecipitated, click-labeled and probed as in (Fig. 1C).

(**E**) Whole cell lysate of 293T cells was fractionated by sucrose gradient ultracentrifugation. Rpt2 myristoylation and proteasome subunits in each fraction were analyzed by western blot.

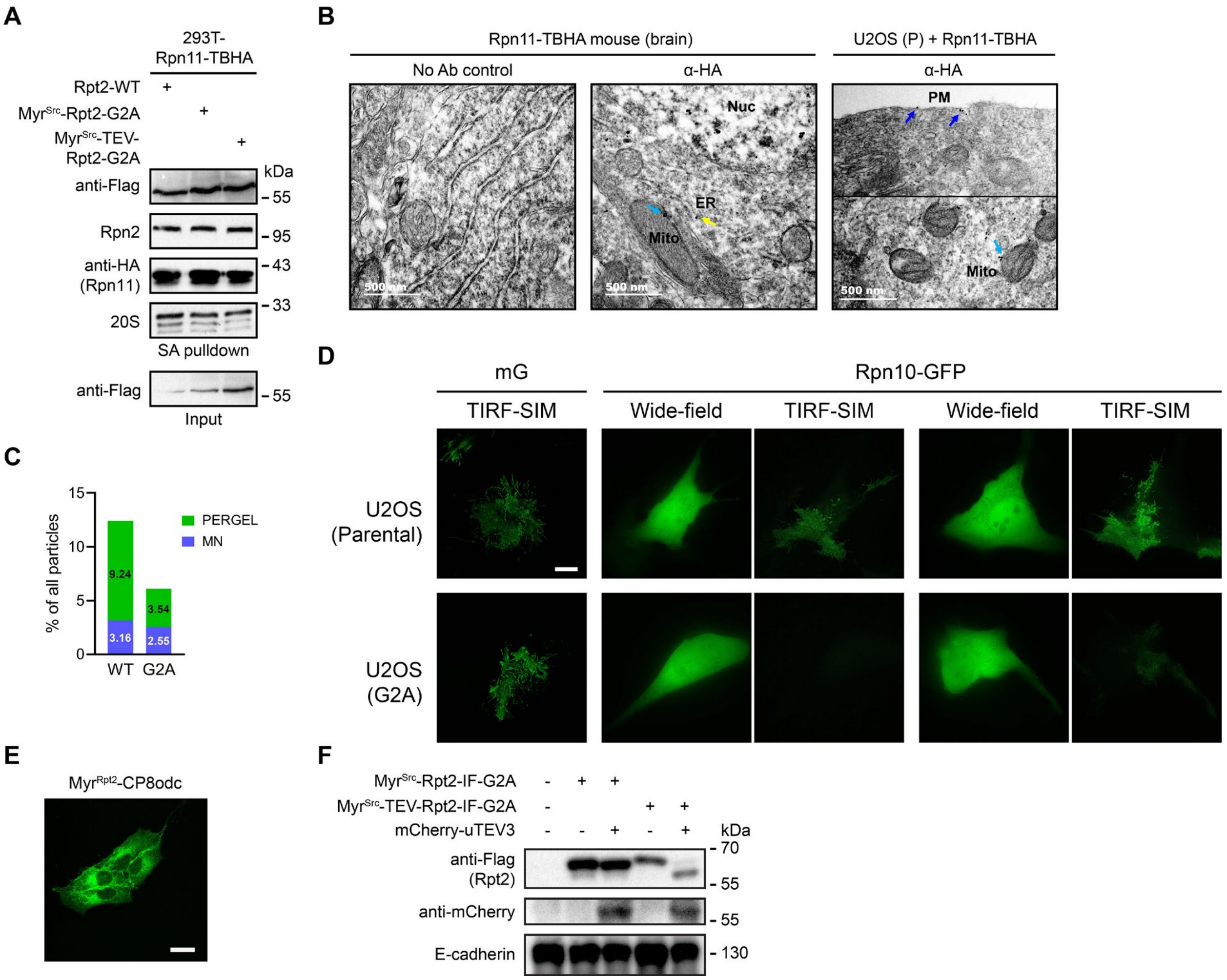

fig. S2. The role of Rpt2 myristoylation in determining proteasome membrane localization.

(**A**) 293T-Rpn11-TBHA stable cells were transfected with the indicated Rpt2-IF variants. Their incorporation into the 26S proteasome was confirmed by streptavidin pulldown and anti-Flag immunoblotting.

(**B**) Representative IEM images from the indicated cell/tissue samples stained with or without anti-HA antibody. Nuc, nucleus; ER, endoplasmic reticulum; Mito, mitochondrion; PM, plasma membrane.

(**C**) Quantification of membrane-associated gold particles (26S proteasomes) in WT and G2A cells. All micrographs from both cell types (MEF and U2OS) with both antibodies (anti-HA and anti-PSMA2) were taken into account. Rpt2-G2A mutation mainly reduced proteasome association with the plasma membrane, ER, Golgi apparatus, endosome/lysosome compartments ("PERGEL"), but less so with regard to proteasomes at the mitochondria or nuclear envelope ("MN").

(**D**) U2OS (parental and G2A) cells grown on coverslips were transfected with mG or Rpn10-GFP, fixed and imaged with SIM under wide-field or TIRF mode. Scale bar = 10 μm (X = Y = 2,048 pixels, 32.5 nm/pixel). The mG construct has the MGCCFSKT sequence fused to the N-terminus of EGFP and marks the plasma membrane.

(**E**) A single clone of U2OS cells stably expressing the Myr^{Rpt2}-CP8odc reporter were imaged by a con-focal fluorescence microscope. Scale bar = 20 µm.
(**F**) The indicated Myr^{Src}-Rpt2 variants were co-transfected with mCherry-uTEV3 into 293T cells. Cleavage of Myr^{Src}-TEV-Rpt2-G2A was confirmed by western blot.

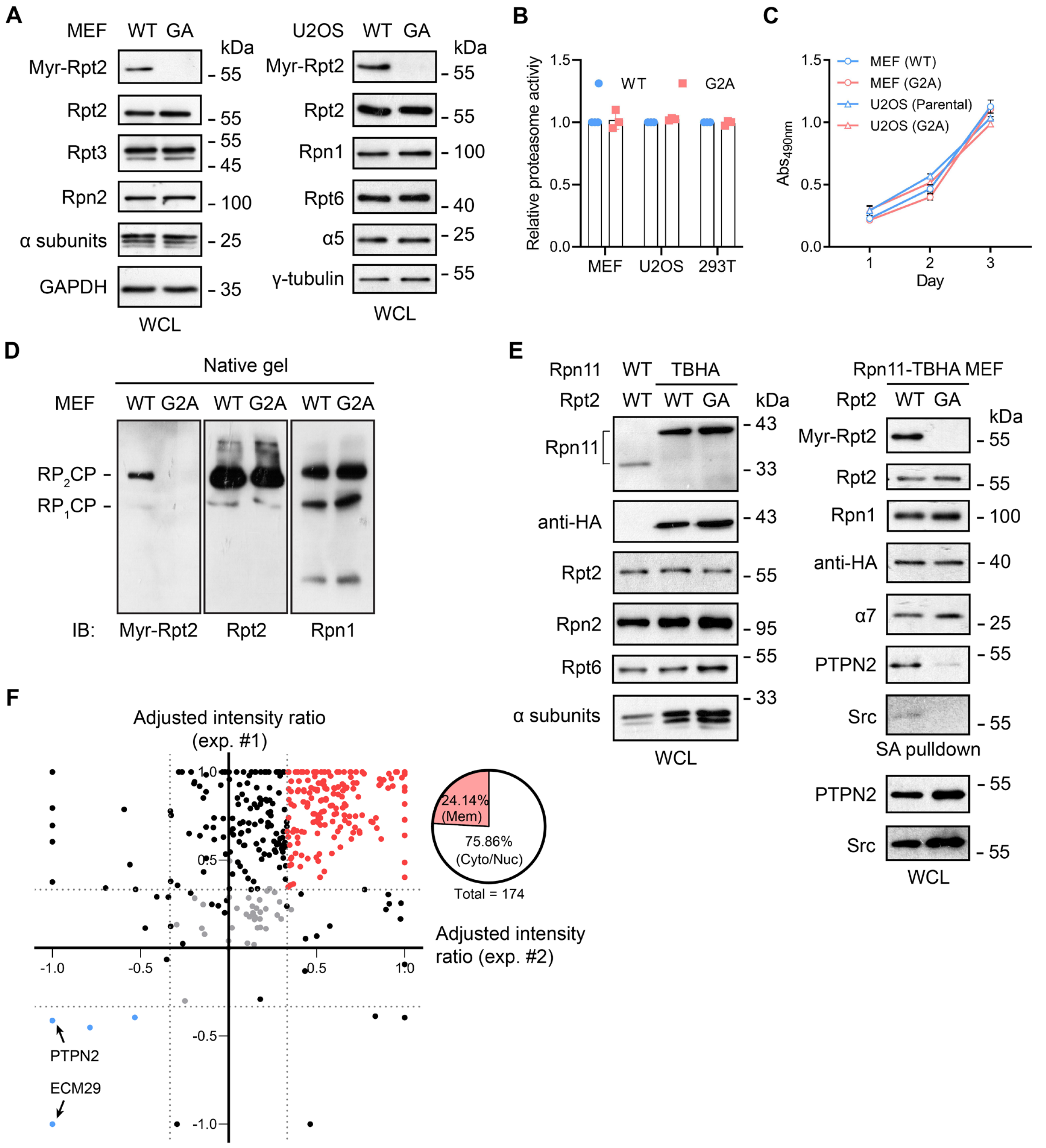

fig. S3. Effects of Rpt2-G2A mutation on proteasome assembly, activity, interactome and cell viability.

(**A**) Western blot analysis of proteasome subunits in WT and G2A cells.
(**B**) Rpt2-G2A knock-in (MEFs, U2OS cells) or replacement (293T pLL3.7 cells) did not affect proteasome peptidase activity against the fluorigenic peptide substrate Suc-LLVY-AMC ($N = 3$).
(**C**) Viability and proliferation of the indicated cells were measured by the CCK-8 assay ($N = 3$).
(**D**) Equal amounts of whole cell lysates from WT and G2A/G2A MEFs were resolved by native PAGE and probed with the indicated antibodies.

(**E**) Western blot analysis showing equal abundance (left) and assembly (right) of the proteasome in Rpt2$^{+/+}$;Rpn11$^{TBHA/TBHA}$ and Rpt2$^{G2A/G2A}$;Rpn11$^{TBHA/TBHA}$ MEFs. Proteasome-bound PTPN2 and Src were also probed.

(**F**) Rpt2-G2A mutation alters the proteasome interactome. Endogenous proteasomes were streptavidin-purified from Rpt2$^{+/+}$;Rpn11$^{TBHA/TBHA}$ and Rpt2$^{G2A/G2A}$; Rpn11$^{TBHA/TBHA}$ MEFs and analyzed by label-free mass spectrometry. Proteasome-interacting proteins (PIPs) identified from two independent experiments were plotted by their adjusted intensity ratios between WT and G2A cells. The adjusted intensity ratio was calculated as $(Intensity_{G2A} - Intensity_{WT})/(Intensity_{G2A} + Intensity_{WT})$, such that a two-fold increase or decrease in G2A cells corresponds a ratio of 0.33 or -0.33, respectively (marked by dotted lines). By this standard, a total of 174 PIPs consistently showed increased proteasome binding in G2A cells (red), over 75% of which were cytosolic or nuclear (non-membrane-associated) proteins. Four PIPs showed decreased binding (blue), including PTPN2 and Ecm29 (arrows). Core proteasome subunits (grey) remained essentially unchanged. Other PIPs are labeled black.

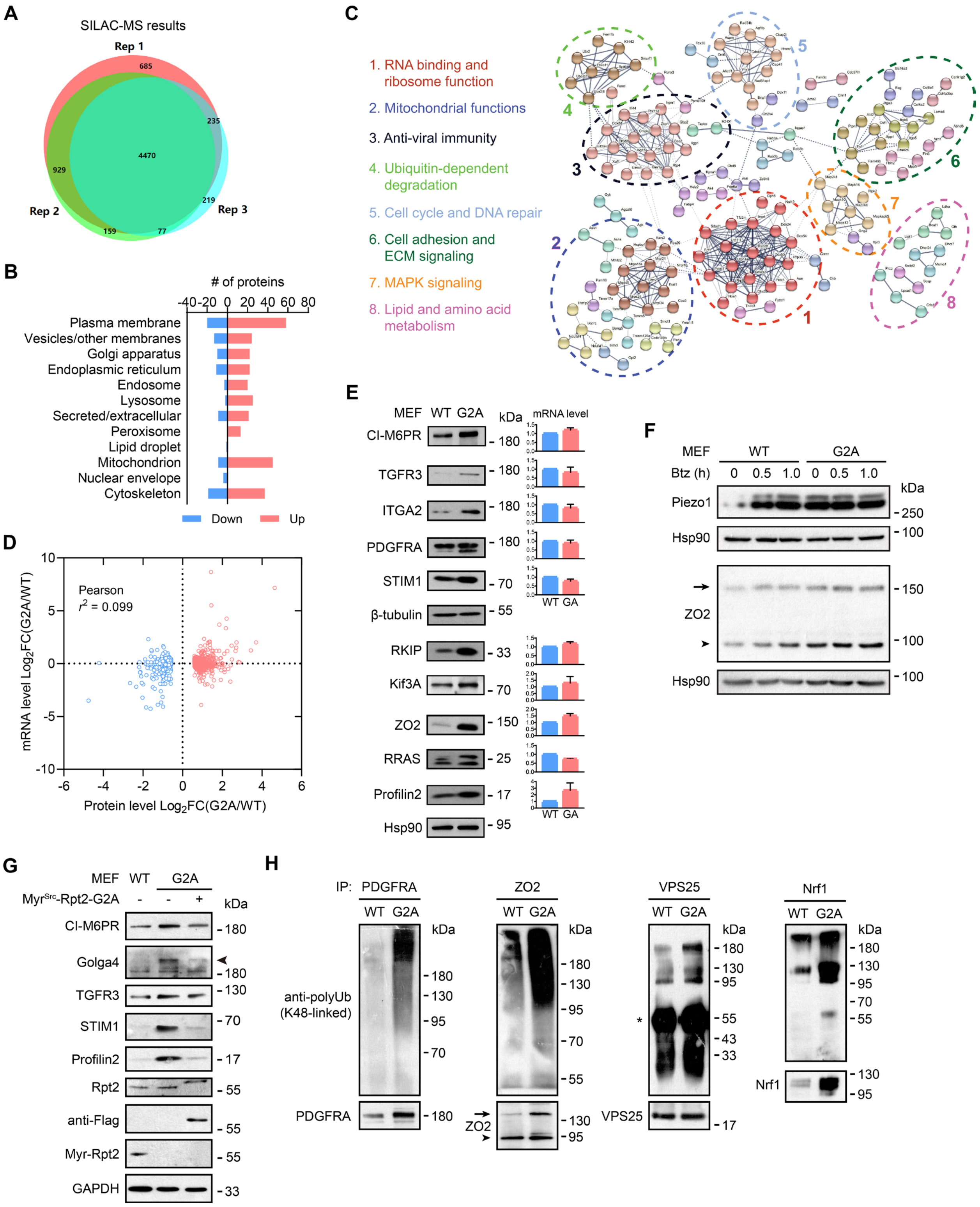

fig. S4. Changes in the membrane proteome in Rpt2-G2A cells.

(**A**) Venn diagram showing the numbers of proteins identified from WT and G2A MEFs in three SILAC-MS experiments.

(**B**) Subcellular distribution of proteins up- and down-regulated in G2A MEFs.

(**C**) Protein-protein interaction network of downregulated proteins in G2A MEFs.
(**D**) Correlation analysis between the protein and mRNA levels of upregulated (red) and downregulated (blue) proteins in G2A cells, based on SILAC-MS and RNA-Seq data.
(**E**) Western blot verification of upregulated transmembrane and peripheral membrane proteins that were identified by SILAC-MS. The corresponding mRNA levels from RNA-Seq analysis are shown on the side ($N = 3$).
(**F**) WT and G2A MEFs were treated with Btz (0.5 μM) for the indicated time and probed for PIEZO1 and ZO2. The arrow and arrowhead indicate full-length and a shorter isoform of ZO2, respectively. Hsp90 was shown as loading control.
(**G**) Myr^{Src}-Rpt2-G2A expression in G2A MEFs restored the levels of the indicated proteins.
(**H**) The indicated proteins were immunoprecipitated from WT and G2A MEFs under denaturing conditions and probed for polyubiquitination. The arrow and arrowhead indicate full-length and the shorter isoform of ZO2, respectively. *, heavy chain of the IP antibody. Each experiment was repeated twice, and representative blots are shown.

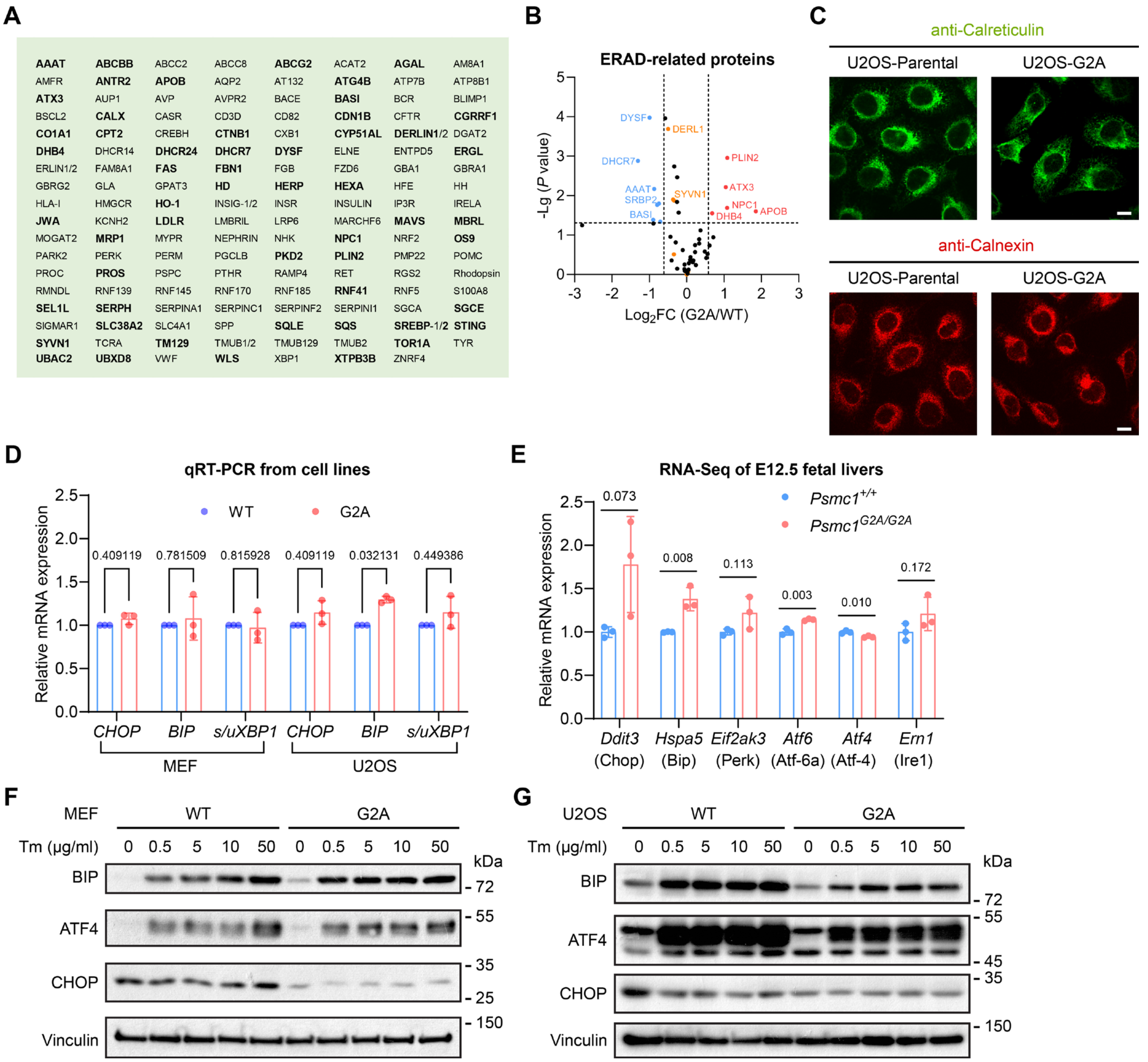

fig. S5. Rpt2-G2A mutation does not cause gross ER stress.

(**A**) A partial list of known ERAD substrates and ERAD-related E3 ligase components (refs. 40, 41). Proteins identified in our SILAC-MS experiments are highlighted in bold.

(**B**) Volcano plot of identified ERAD-related proteins as shown in (A). ERAD substrates significantly up- and down-regulated in G2A cells are marked red and blue, respectively ($P <$ 0.05). Orange dots indicate E3 ligase components of the ERAD pathway.

(**C**) Immunofluorescence staining of the indicated ER markers in U2OS cells. Scale bar = 10 µm.

(**D**) qRT-PCR analysis of ER stress/UPR-related genes in MEFs and U2OS cells. For *XBP1*, the spliced/unspliced (s/u) ratios are plotted. *P* values are shown (Student's *t*-test, two-tailed, $N = 3$).

(**E**) RNA-Seq results of the indicated ER stress/UPR-related genes in fetal liver tissues from E12.5 WT and G2A embryos. The names of the corresponding protein produces are listed below in parentheses. *P* values are shown (Student's *t*-test, two-tailed, $N = 3$ pairs of embryos).

(**F, G**) Increasing concentrations of tunicamycin (Tm) were used to treat MEFs (F, 10 hours) and U2OS cells (G, 8 hours). Total cell lysates were probed for the indicated ER stress-related proteins.

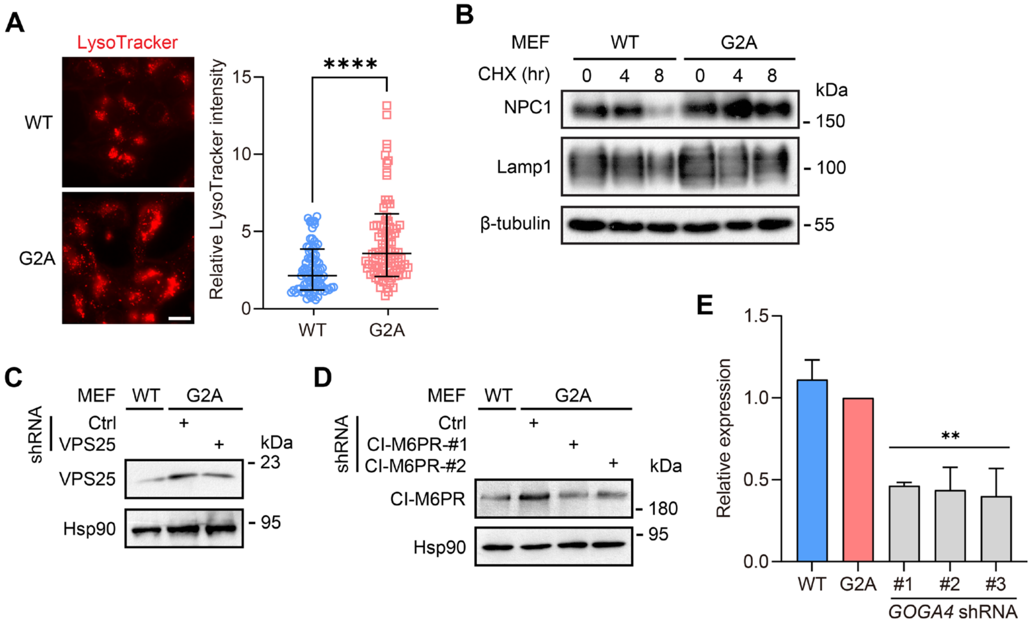

fig. S6. Analysis of lysosome contents.

(**A**) LysoTracker staining of U2OS cells. Scale bar = 10 μm. ****, $P < 0.0001$ (two-tailed *t*-test, unpaired. WT, $N = 83$; G2A, $N = 96$).

(**B**) WT and G2A MEFs were treated with cycloheximide (CHX, 50 μg/ml) for the indicated time. Endogenous NPC1 and Lamp1 were probed.

(**C, D**) MEFs were infected with lentiviruses of the pSuya-GFP backbone expressing VPS25 (C) or CI-M6PR (D) shRNAs. After GFP enrichment, cells were analyzed for knockdown efficiency by western blot.

(**E**) MEFs were infected with 3 independent shRNAs targeting *GOGA4* as in (C, D) and the mRNA level of *GOGA4* was analyzed by qRT-PCR. **, $P < 0.01$ (Student's *t*-test, two-tailed, $N = 3$)

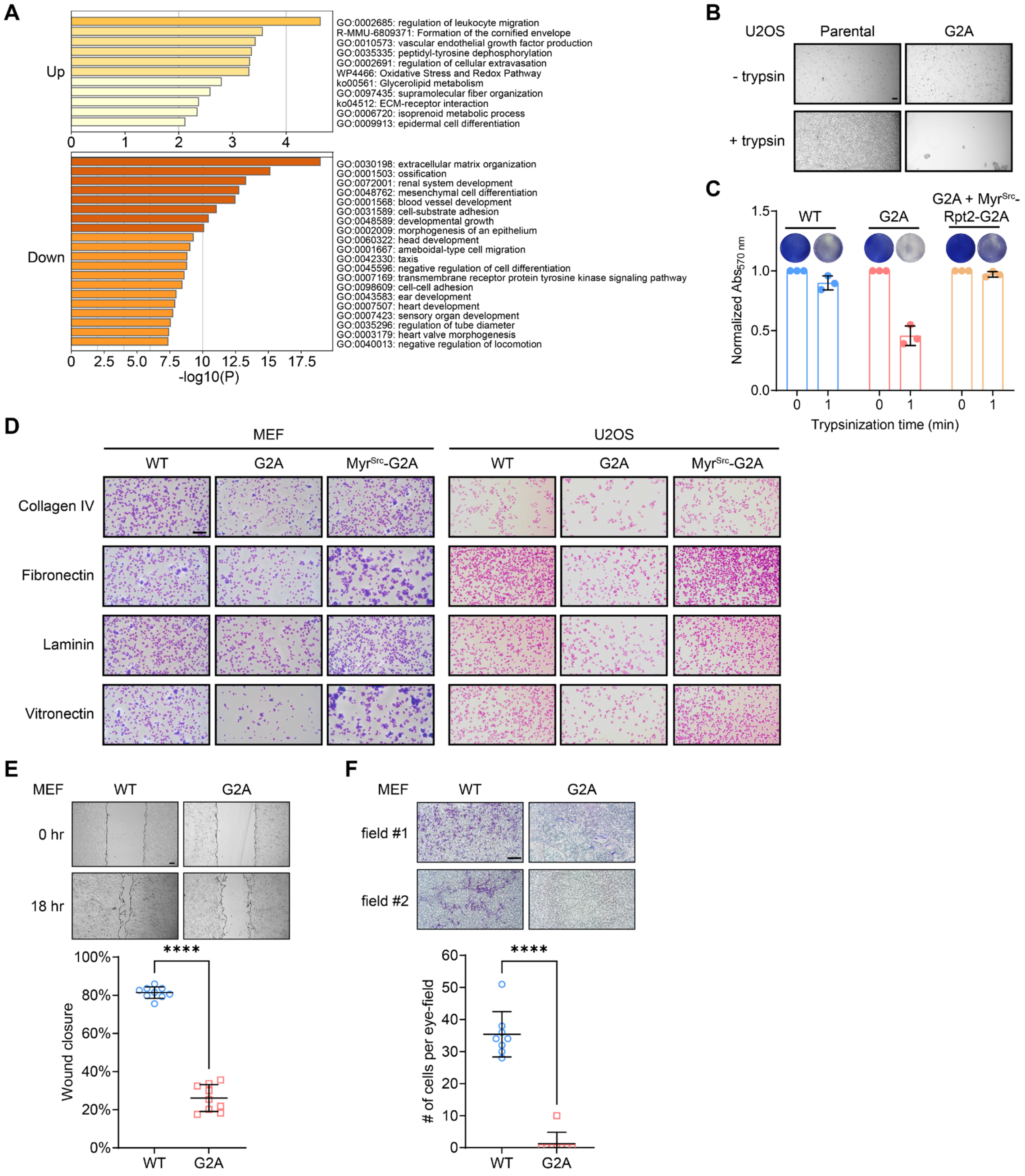

fig. S7. Loss of Rpt2 myristoylation undermines cell adhesion and migration.

(**A**) GO term analysis of differentially expressed mRNAs identified in G2A MEFs by RNA-Seq.
(**B**) U2OS cells were seeded in a 12-well plate at 1.0 x 10^5/well. The next day, cells were washed with PBS and briefly treated with 0.05% trypsin/EDTA at 37℃ for 2 min. Photographs were taken before and after trypsinization. Scale bar = 100 µm.
(**C**) The indicated MEFs were treated without or with trypsin/EDTA (0.0625%, 1 min). Cells were fix-stained with crystal violet immediately after 1-min treatment of trypsin and imaged.

After extensive washes, the amounts of crystal violet retained were quantified by measuring absorbance at 570 nm (*N* = 3).
(**D**) Representative photographs of crystal violet-stained cells adhered to ECM protein-coated surfaces. Scale bar = 100 µm.
(**E**) WT and G2A MEFs were allowed to migrate for 18 hours in a scratch/wound-healing assay. Reduction of the wound area was measured and plotted. Scale bar = 100 µm. [****], $P < 0.0001$ ($N = 9$ eye-fields from 3 independent experiments, Student's *t*-test).
(**F**) Representative images of porous membranes of the transwell migration assay, with migrated MEFs stained with crystal violet. Scale bar = 100 µm. The numbers of migrated cells in each eye-field were counted and plotted. [****], $P < 0.0001$ ($N = 8$, Student's *t*-test).

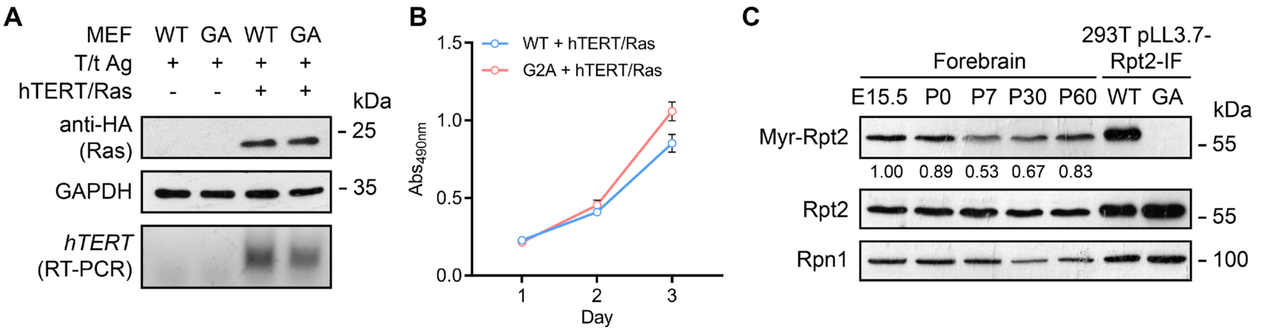

fig. S8. Oncogene expression in MEFs and Rpt2 myristoylation during brain development.
(**A**) Stable expression of N-Ras^{G12V} and hTERT in immortalized MEFs was confirmed by western blot (top) and RT-PCR (bottom), respectively.
(**B**) Proliferation of oncogene-transformed MEFs was measured by the CCK-8 assay ($N = 3$).
(**C**) The dynamics of Rpt2 N-myristoylation level in rodent forebrain from embryonic stage to adulthood was determined by western blot. The ratios between Myr-Rpt2 and total Rpt2 at each developmental stage are shown.

Additional supplemental auxiliary files:

Table S1. PIPs in WT and G2A MEFs.
Table S2. SILAC - Overlap of 3 biological repeats
Table S3. SILAC - Differentially regulated proteins
Table S4. Localizations of differentially regulated proteins
